# Single-Cell Membrane-Permeabilization Kinetics Reveal Species-Specific Responses to Antimicrobial Photodynamic Treatment

**DOI:** 10.1021/acsomega.6c09003

**Published:** 2026-09-15

**Authors:** Omnia Ahmed, Sol R. Martínez, Taufiq Khan, Nitika Paudyal, Yusra Amena, Mahmoud Abouelyazid, Andrés M. Durantini

**Affiliations:** † Department of Chemistry, 33140 Southern Illinois University Edwardsville, Edwardsville, Illinois 62026, United States; ‡ National Corn to Ethanol Research Center, Southern Illinois University Edwardsville, Edwardsville, Illinois 62025, United States; § Department of Electrical and Computer Engineering, 14736 Texas A&M University, College Station, Texas 77843, United States

## Abstract

Bacterial populations that appear uniform can hide striking differences in how individual cells respond to antimicrobial stress. This hidden heterogeneity is especially important for antimicrobial photodynamic treatment (aPDT), where population-averaged assays report whether bacteria recover but cannot reveal when individual cells become damaged or why some cells respond later than others. Here, we developed an agarose-pad single-cell imaging platform to track membrane permeabilization during aPDT mediated by Br_2_B, a brominated boron dipyrromethene (BODIPY) dye. Using extraintestinal pathogenic *Escherichia coli* UMN026 and *Staphylococcus aureus* SA113 as model pathogens, time-resolved propidium iodide (PI) fluorescence trajectories were fitted for individual bacteria to extract the onset of PI entry (*t*_i_), final PI-positive transition time (*t*_f_), and transition duration (Δ*t* = *t*_f_ – *t*_i_). This approach revealed distinct species-specific response architectures that were not apparent from bulk measurements alone. In *E. coli*, photodynamic response heterogeneity was distributed across both delayed PI-entry onset and prolonged transition duration, particularly under nutrient-rich LB conditions and in mixed-founder populations. In contrast, *S. aureus* displayed a compressed onset phase, with heterogeneity emerging mainly after PI entry had begun. Br_2_B uptake was consistently higher in *S. aureus* than in *E. coli*, supporting a model in which envelope architecture and photosensitizer access determine where heterogeneity appears during photodynamic damage. Post-aPDT regrowth assays further showed that recovery kinetics were species- and population-dependent, linking single-cell membrane-permeabilization dynamics with later population-level outgrowth. Finally, mutation-rate estimates indicated that colony-derived inocula are not genetically identical, but that mutation-derived diversity is unlikely to be the dominant source of the observed timing patterns. Together, these results demonstrate that aPDT response is not a single synchronized event but a structured, species-dependent process shaped by photosensitizer access, nutrient context, founder-lineage history, and physiological heterogeneity. This work establishes single-cell PI-entry kinetics as a powerful framework for uncovering hidden antimicrobial response architectures during photodynamic treatment.

## Graphical abstract

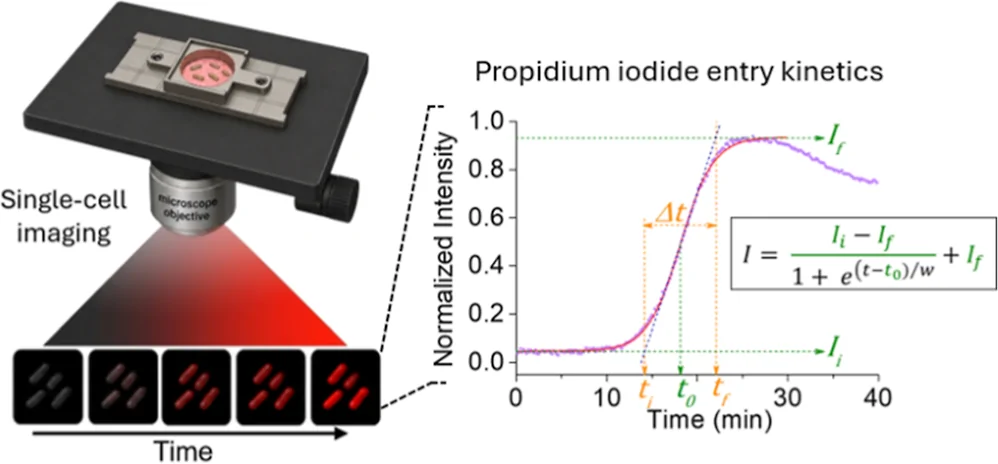

## 1 Introduction

Localized bacterial infections, including acute and chronic wound infections, remain difficult to treat because bacteria can persist in heterogeneous microenvironments that differ in nutrient availability, oxygen tension, biofilm structure, host-derived stress, and antimicrobial exposure.^1,2^ These spatial and physiological gradients can allow subpopulations of cells to delay or withstand antimicrobial stress even when the population is derived from a pure isolate.^3,4^ Thus, understanding antimicrobial outcome requires not only measuring whether a bacterial population survives, but also determining how individual cells progress through damage and inactivation over time.

Antimicrobial photodynamic treatment (aPDT) is a promising strategy for localized, topical, and surface-associated infections because it combines a photosensitizer (PS), visible light, and molecular oxygen to generate cytotoxic reactive oxygen species such as singlet oxygen (^1^O_2_).^5–7^ Unlike many antibiotics that act on defined molecular targets, aPDT produces a broad oxidative effect capable of damaging membranes, proteins, lipids, and nucleic acids simultaneously.^8^ This multi-target mechanism reduces the likelihood that a single genetic change will provide genetic elements to resist the treatment. However, it does not eliminate the possibility that bacterial subpopulations differ in PS uptake, envelope permeability, oxidative-stress tolerance, repair capacity, or physiological state.

Bacterial heterogeneity is recognized as a determinant of antimicrobial outcome. Cells within a genetically related population can differ in metabolic activity, envelope composition, redox state, stress-response activation, and growth phase. Such non-genetic variability can generate persister-like or transiently tolerant subpopulations that survive antimicrobial exposure without requiring stable heritable resistance.^3,4,9,10^ This is especially relevant for aPDT, because bacterial survival depends on both the timing of oxidant exposure and the capacity of individual cells to limit, buffer, or repair damage.

At the same time, complete genetic uniformity should not be assumed for colony-derived inocula. Each visible colony expands from a founder cell through repeated replication cycles, and spontaneous replication errors that escape proofreading and mismatch repair can generate low-frequency mutant sublineages during colony growth.^11–14^ Therefore, single-colony-derived populations are best considered highly related founder-lineage populations rather than perfectly uniform cultures. Mixing colonies or collecting cells from confluent streak regions further increases the number of independent founder-lineage histories represented in the inoculum. This distinction is important for interpreting population structure, although mutation-derived heterogeneity is expected to represent only a minority component relative to physiological heterogeneity.

Most studies of antimicrobial heterogeneity have focused on antibiotics, whereas less attention has been given to how single-cell variability shapes responses to photodynamic oxidative stress. This gap is important because bulk aPDT assays report population-averaged outcomes and can bias whether individual cells have prolongated survival time when exposed to the treatment. Microscopy provides a reliable toolbox for real-time imaging the viability of living bacteria. Ortega et al. monitored Min protein system to report changes in bacterial physiology. They found that bacterial responses to aPDT depend strongly on the local mode of PS delivery. In *Escherichia coli* exposed to methylene blue loaded PEGDA hydrogels, reversible PS loading produced rapid and, in many cells, abrupt loss of Min-protein oscillations, whereas irreversible immobilization of the PS caused a more gradual slowing of these oscillations.^15^ Barroso et al. developed a spatiotemporally resolved single-cell platform to study how the distance from a localized ROS source influences bacterial photoinactivation. Using *Staphylococcus aureus* positioned around phthalocyanine-functionalized microparticles, authors demonstrated that cells in direct contact with the PS source showed propidium iodide (PI) uptake and membrane damage substantially earlier than cells located several micrometers away. This finding indicates that individual cells at comparable distances still displayed variable inactivation times, demonstrating that both local ROS exposure and intrinsic cell-to-cell heterogeneity influence photodynamic responses.^100^

We have shown that time-resolved fluorescence microscopy can reveal substantial cell-to-cell variation during aPDT, with individual bacteria from the same culture showing markedly different PI-entry and inactivation times.^16^ Building on this foundation, the present study examines two pathogenic bacterial strains and evaluates how species identity, nutrient availability, and founder-lineage population structure influence the kinetics of photodynamic damage. A key improvement was the use of an updated microscope system with a higher sensitivity camera and the development of a custom 3D-printed sample holder containing an agarose-pad chamber, in which bacteria were immobilized. Unlike the previous glass adhesion-based configuration, this updated setup maintained the position of individual cells during prolonged illumination and time-lapse imaging, while reducing the potential sampling bias associated with preferential retention of adhesion-prone cells.

In this study, we compared the extraintestinal pathogenic (ExPEC) Gram-negative strain *E. coli* UMN026 with the Gram-positive opportunistic pathogen *S. aureus* SA113. ExPEC strains are major concern due to urinary tract infections, bacteremia, sepsis, and neonatal meningitis, whereas *S. aureus* commonly causes skin and soft-tissue infections, wound infections, pneumonia, endocarditis, bloodstream infections, and device-associated infections.^11,17–20^ The two bacterial species also differ substantially in cell-envelope structure: *E. coli* has an outer membrane that can restrict PS access, whereas *S. aureus* lacks this barrier and has a thick, porous peptidoglycan layer.^7,8,21–23^ Cells were studied in PBS and in nutrient-rich media (LB for *E. coli* and TS for *S. aureus*) to evaluate the effect of nutritional environments. A brominated boron dipyrromethene (BODIPY) dye, Br_2_B, was selected as the PS. Br_2_B is photostable and has a high singlet oxygen quantum yield (84%).^5^ In addition, we have used this PS in previous studies, which allows comparison with previous work. Br_2_B-mediated aPDT was monitored by time-lapse PI fluorescence microscopy. The goal of this study was not to compare different PSs, but to test how bacteria with different cell envelopes develop single-cell PI-entry heterogeneity under the same aPDT treatment in different media. Because PI becomes fluorescent after entering cells with damaged envelopes and binding nucleic acids, the measurements reflect the timing of envelope permeabilization rather than an exact time of cell death.^24,25^ Individual isolate populations were obtained from single colonies, whereas the combined population was collected from the confluent growth region and therefore contained multiple founder lineages. For each cell, we measured the onset of PI entry (*t*_i_), the final PI-positive time (*t*_f_), and the transition duration (Δ*t* = *t*_f_ – *t*_i_). We hypothesized that single-cell timing heterogeneity would be distributed differently in the two species, reflecting their distinct envelope architectures and PS access, and that comparing single-colony-derived isolates with combined, multi-founder populations would help distinguish physiological from founder-lineage and mutation-derived sources of variability.

Our results show that *E. coli* and *S. aureus* differ not only in their overall response to photodynamic stress, but also in the timing and extent of heterogeneity during envelope damage.

## 2 Results and Discussion

### 2.1 Imaging Platform for Stable Single-Cell Photodynamic Analysis

Stable long-term imaging was required to quantify bacterial survival dynamics during aPDT at single-cell resolution. In our previous configuration,^16^ analysis was restricted to cells that remained adhered to the glass surface. This created potential sampling bias because cells that detached from the coverslip moved out of the focal plane, and adhesion-prone cells may not represent the full physiological diversity of the population.

To address these limitations, we designed and 3D-printed a custom sample holder incorporating a central agarose-pad chamber (Figure 1). Bacteria were immobilized in a sandwich geometry between the agarose pad and the coverslip. The agarose pad can be supplemented with the desired medium, Br_2_B, PI, and other fluorescent reporters, allowing defined microenvironments to be maintained while preserving the position of individual cells during illumination and imaging.

**Figure 1:**
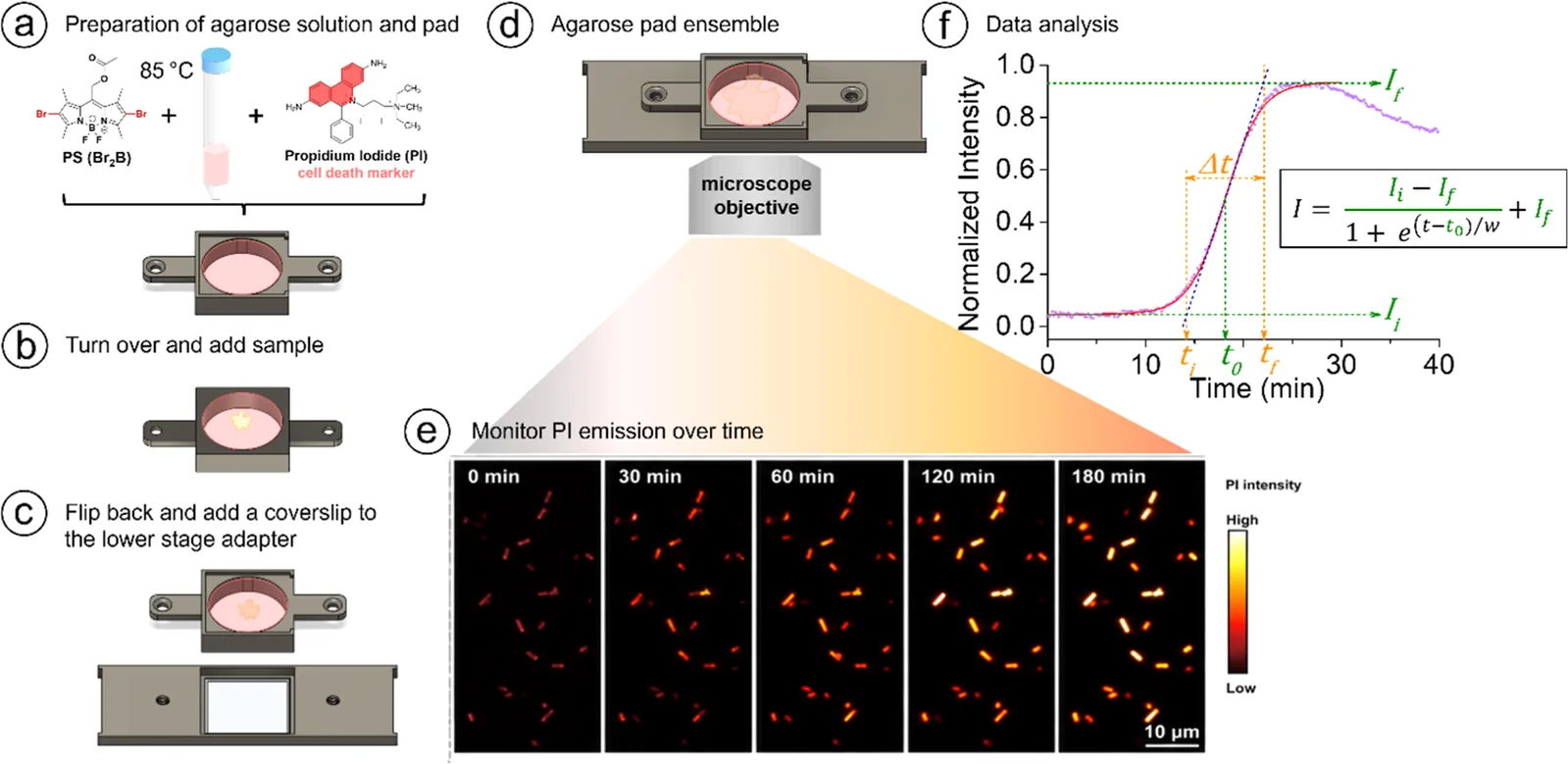
Agarose-pad-based imaging platform for time-lapse single-cell analysis during aPDT. (a) Agarose solutions were prepared by mixing the PS (Br_2_B) and/or PI in the desired medium. The solution was then poured into the chamber and allowed to cool to room temperature. (b) The agarose pad was flipped over and a bacterial suspension was placed on top of the pad. (c) The chamber was returned to its original position and fitted into the sample holder, creating a sandwich configuration in which the bacteria were positioned between the agarose pad and the coverslip. (d) During illumination, PI fluorescence was monitored over time to follow progressive membrane permeabilization at the single-cell level. (e) Representative images show increasing PI emission from individual bacteria at 0, 30, 60, 120, and 180 min. (f) Single-cell PI fluorescence trajectories were fitted with a sigmoidal Boltzmann function. *I*_i_ and *I*_f_ represent the fitted initial and final fluorescence intensities, respectively; *t*_0_ represents the midpoint of the sigmoidal increase; and *w* represents the fitted Boltzmann width parameter. The onset time of PI uptake (*t*_i_) and final PI fluorescence time (*t*_f_) were determined from the intersections between the tangent at *t*_0_ and the fitted *I*_i_ and *I*_f_ levels, respectively. The PI-uptake duration was then calculated as Δ*t* = *t*_f_ – *t*_i_.

Bacterial responses to aPDT were quantified by tracking PI fluorescence at the single-cell level. PI is a membrane-impermeant nucleic acid stain commonly used as a reporter of compromised envelope integrity. In cells with intact envelopes, PI is largely excluded; after membrane damage or permeabilization, PI can enter the cell, bind nucleic acids, and increase emission intensity. Because PI fluorescence can overestimate loss of viability in some settings, including adherent bacterial cells, biofilms containing extracellular nucleic acids, or cells with altered membrane potential,^24,25^ brightfield images were acquired in parallel with fluorescence images to confirm bacterial inactivation, position, morphology, and single-cell identity (Figures S1 and S2). Control experiments were performed to confirm that PI fluorescence changes required both Br_2_B and green light irradiation. For *E. coli*, green-light exposure in the absence of Br_2_B produced only minimal PI fluorescence after 6 h of irradiation, corresponding to a total radiant exposure of 14.3 ± 0.4 J/cm^2^ (Figure S3). Similarly, *E. coli* incubated with Br_2_B in the dark for 6 h showed PI fluorescence in less than 5% of the bacterial count in the field of view, indicating that Br_2_B alone did not produce substantial membrane permeabilization under these conditions (Figure S4). The same control design was applied to *S. aureus*. Only a few cells (<6%) showed fluorescence in the PI channel after exciting with green for 2 h in the absence of the PS (light dose: 4.75 ± 0.14 J/cm^2^) (Figure S5). Furthermore, Br_2_B incubation in the dark for 2 h did not produce substantial PI staining (Figure S6). These controls, performed using agarose pads prepared with PBS, confirm that the observed PI emission requires Br_2_B-mediated photodynamic activity.

For each bacterium, a region of interest was defined and used to extract the mean PI fluorescence intensity throughout the image sequence (Figure 1f). The resulting trajectories were fitted with a sigmoidal Boltzmann model. The fit provided the initial fluorescence intensity (*I*_i_), final fluorescence intensity (*I*_f_), midpoint time (*t*_0_), and fitted Boltzmann width parameter (*w*). The onset and final times of PI uptake were then determined from the fitted curve using a tangent-intersection method. Specifically, *t*_i_ was defined as the intersection between the tangent at *t*_0_ and the fitted initial fluorescence level (*I*_i_), whereas *t*_f_ was defined as the intersection between the same tangent and the fitted final fluorescence level (*I*_f_). The PI-uptake duration was calculated as Δ*t* = *t*_f_ – *t*_i_ = 4w. Thus, *t*_i_, *t*_f_, and Δ*t* were extracted from the fitted trajectory rather than from arbitrary fluorescence-intensity thresholds.

This analytical framework was applied to single colony derived populations (isolates) and combined populations of *E. coli* and *S. aureus* under PBS and nutrient-rich conditions. Each isolated population was prepared from a separate well-isolated colony, whereas the combined population was prepared by collecting cells from the confluent streak region and therefore contained cells from multiple colony-derived founder lineages (Figure S7). *E. coli* was evaluated in PBS and LB, whereas *S. aureus* was evaluated in PBS and TS. These comparisons enabled species identity, medium composition, and population structure to be evaluated under the same photodynamic oxidative challenge.

### 2.2 Distribution of Onset and Transition Heterogeneity Across Conditions

PBS was used as a defined, nutrient-deficient reference condition to reduce the chemical and physiological complexity introduced by nutrient-rich media and to provide a baseline for comparing single-cell responses. However, nutrient deprivation itself can induce stress adaptation and therefore does not eliminate physiological-state heterogeneity.^26^ In contrast, LB and TS provide nutrients that support growth and can alter bacterial metabolism, biomass accumulation, and envelope-associated properties.^27–29^ In *S. aureus*, growth environment has been shown to modify membrane fatty-acid composition, a property that can influence membrane behavior and susceptibility to oxidative or membrane-active stress.^29^ In addition, extracellular medium components can directly modify photodynamic efficacy; for example, albumin and plasma reduced photodynamic inactivation relative to PBS in several microorganisms.^30^

Box plots were used to determine whether cell-to-cell variability occurred mainly in *t*_i_, Δ*t*, or *t*_f_. The lower and upper edges of each box define the 25th and 75th percentiles, respectively; therefore, broad boxes indicate that heterogeneity is present across the central 50% of cells rather than only in a few outliers. The empty square marks the mean, which helps identify conditions in which a subset of highly delayed cells shifts the population average upward relative to the median. In addition, the lines or whiskers extending from each box indicate the range of values observed within each population. Because isolate 1, isolate 2, isolate 3, and the combined population were each prepared from independent founder lineages within the same species and medium, the spread among these four samples is itself a measure of heterogeneity, complementary to the cell-to-cell spread captured within each box. Both axes of variability -among single-colony derived populations, and among cells within a single population- are addressed quantitatively in Section 2.5.

A clear species-dependent difference in susceptibility was observed. *E. coli* required substantially longer exposure times to undergo membrane permeabilization than *S. aureus*. Across the conditions analyzed, the within-population *t*_i_ ranges were 104.0–322.5 min for *E. coli* and 5.1–15.7 min for *S. aureus*, whereas the corresponding *t*_f_ ranges were 180.8–425.5 min for *E. coli* and 24.5–73.7 min for *S. aureus* (Figure 2 and Table S1). In PBS, *E. coli* spread was evident in both *t*_i_ and Δ*t*, indicating that cells varied in when detectable PI uptake began and in how long the transition to terminal PI staining lasted. The middle 50% of *t*_i_ values ranged from approximately 23–73 min in isolate 1, 69–123 min in isolate 2, 30–75 min in isolate 3, and 49–136 min in the combined population. The upper and lower whiskers also show that this delayed onset was accompanied by substantial transition durations, with values in the range of 82.3, 95.0, 94.5, and 119.3 min for isolates 1–3 and the combined population, respectively (Figure 2 and Table S1). The same pattern was reflected in *t*_f_, where isolate 2 and the combined population had upper quartiles extending to approximately 190–220 min.

**Figure 2:**
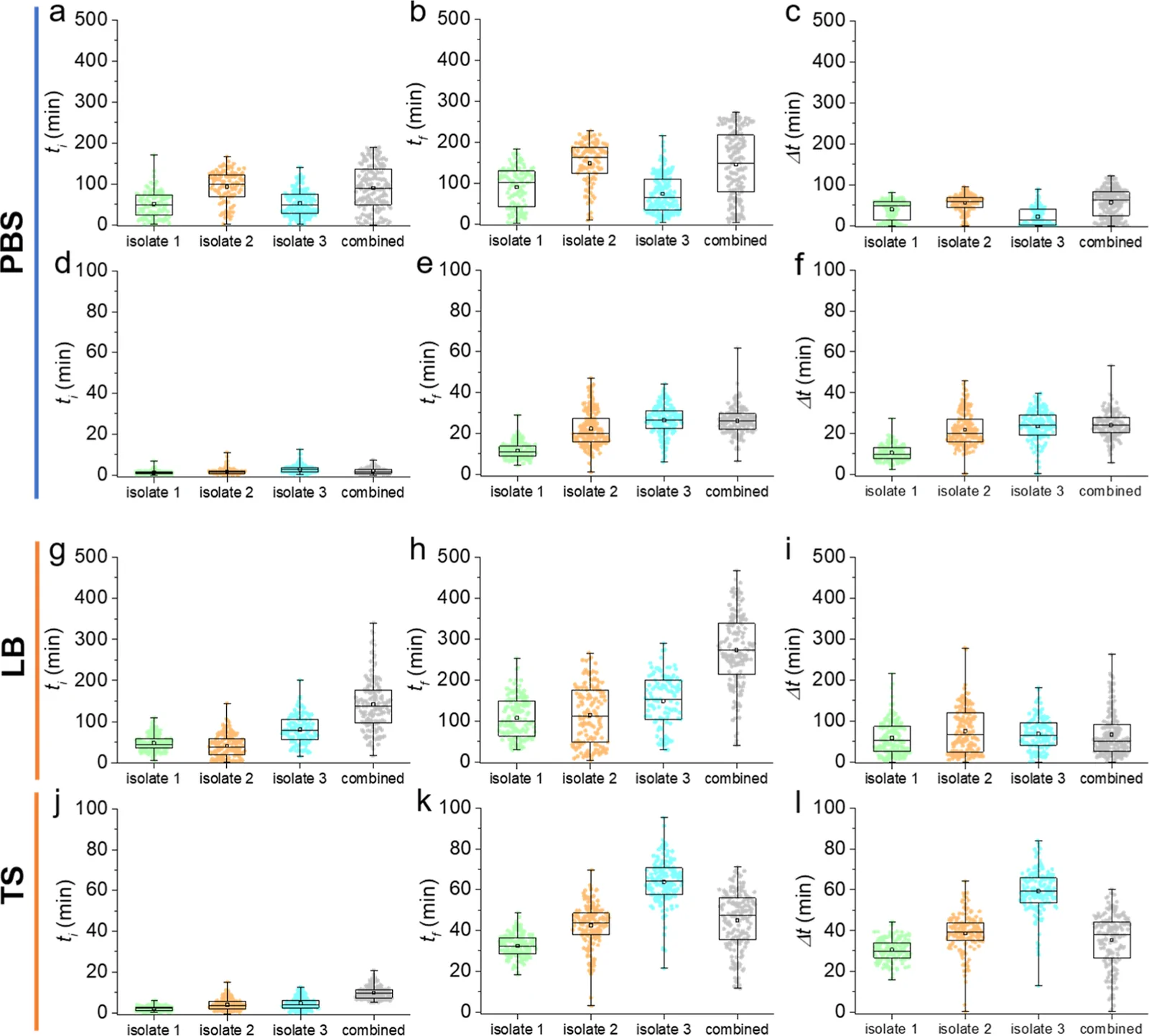
Distribution of single-cell PI-entry kinetic parameters for *E. coli* and *S. aureus*. Box-and-scatter plots show the distributions of *t*_i_, Δ*t*, and *t*_f_ extracted from single-cell PI fluorescence trajectories during the photodynamic treatment. Panels (a–c) show *E. coli* in PBS; panels (d–f), *S. aureus* in PBS; panels (g–i), *E. coli* in LB; and panels (j–l), *S. aureus* in TS. Individual points represent single bacterial cells; boxes represent the interquartile range; the horizontal line indicates the median; the empty square represents the mean; and the lines or whiskers extending from each box indicate the range of values observed within each population. The power of excitation light was 0.66 ± 0.02 mW. Light dose varied between 4.75 ± 0.14 and 14.3 ± 0.4 J/cm^2^.

In *S. aureus* under the same experimental conditions, *t*_i_ was comparatively compressed, whereas Δ*t* and *t*_f_ broadened more strongly across isolates and combined populations. The whiskers values for Δ*t* increased from 26.1 min in isolate 1 to 43.6, 36.5, and 51.3 min in isolates 2, 3, and the combined population, respectively (Figure 2 and Table S1). This pattern indicates that many *S. aureus* cells began detectable PI uptake within a narrower onset window, but diverged after onset in the time required to reach terminal *t*_f_. Therefore, under PBS conditions, *E. coli* showed cell-to-cell variability in both the onset of PI uptake and the duration of PI uptake, whereas *S. aureus* showed variability mainly in the duration of PI uptake after the signal had already begun to increase.

Under nutrient-rich conditions, a similar contrast between species was maintained but expressed through different timing parameters (Figure 2, panels g–l). In *E. coli* grown in LB, the combined population showed a strong upward shift in *t*_i_, with the central 50% of cells distributed approximately between 97 and 177 min. This range was substantially higher than the distributions centered closer to 22–60 min for isolates 1 and 2 and approximately 55–105 min for isolate 3. The whiskers also extended over a broader interval in the combined population, indicating that the shift in *t*_i_ was not limited to the central portion of the distribution but included cells with substantially earlier (17.3 min) and later PI-entry times (339.8 min). The upward shift extended to *t*_f_, where the combined LB population displayed a broad central distribution of approximately 213–337 min. On the other hand, *t*_i_ remained relatively constrained for *S. aureus* grown in TS, whereas Δ*t* and *t*_f_ increased in both central tendency and spread. The Δ*t* interquartile ranges increased from approximately 26–33 min in isolate 1 to 35–44 min in isolate 2, 53–67 min in isolate 3, and 28–44 min in the combined population.

### 2.3 Relationship between *t*_i_, *t*_f_, and Δ*t*

While the box plots show where variability arises within the temporal progression of membrane permeabilization, pairwise comparisons of the kinetic parameters offer a complementary perspective on how *t*_i_, Δ*t*, and *t*_f_ relate to one another within each population.

Plots of *t*_f_ vs *t*_i_ showed that terminal PI fluorescence intensity was delayed relative to the onset of detectable PI entry in every condition (Figure 3). The green line represents the theoretical reference relationship *t*_f_ = *t*_i_. In this case, the final PI fluorescence time would be identical to the onset time of PI uptake, meaning that the transition duration would be zero Δ*t* = *t*_f_ – *t*_i_ = 0. Therefore, the green line is not an experimental fit; it is a reference line showing the limiting case in which PI uptake begins and reaches its final fluorescence plateau instantaneously. The experimental data and red fitted lines are positioned above the green line because, in viable cells, *t*_f_ is greater than *t*_i_. The vertical separation from the green line reflects the magnitude of Δ*t*, or the time required for PI fluorescence to progress from its initial detectable increase to the final plateau.

**Figure 3:**
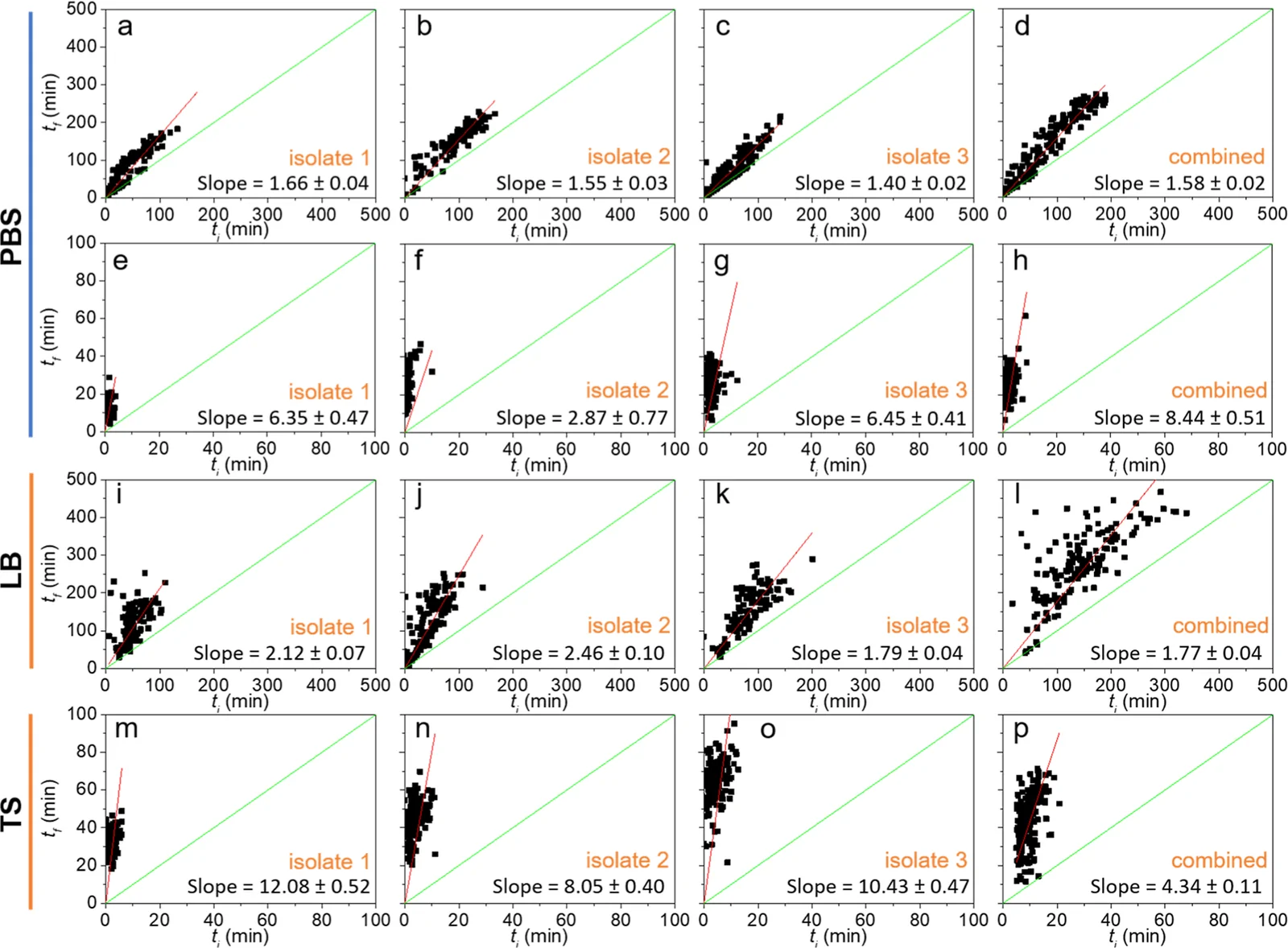
Scatter plots show the relationship between *t*_i_ and *t*_f_ for individual bacterial cells exposed to the photodynamic treatment. Panels (a–d) correspond to *E. coli* in PBS, panels (e–h) to *S. aureus* in PBS, panels (i–l) to *E. coli* in LB, and panels (m–p) to *S. aureus* in TS. Within each condition, the four panels represent isolate 1, isolate 2, isolate 3, and the combined population, respectively. Fitted lines (red) summarize the data, and the green line the case in which Δ*t* = 0. The power of excitation light was 0.66 ± 0.02 mW. Light dose varied between 4.75 ± 0.14 and 14.3 ± 0.4 J/cm^2^.

In *E. coli*, *t*_f_ vs *t*_i_ slopes were moderate, ranging from 1.40–1.66 in PBS and 1.77–2.46 in LB, consistent with a response in which both delayed onset and later transition dynamics shape final timing. In *S. aureus*, the corresponding slopes were higher, ranging from 2.87–8.44 in PBS and 4.34–12.08 in TS, indicating stronger onset-to-final amplification after a constrained onset window. Within each species and medium, the slope also varied considerably among isolates 1–3 themselves: for example, *S. aureus* PBS isolates 1–3 spanned 2.87–6.45 (mean 5.22), a range equal to 69% of the isolate mean; comparable in magnitude to the further shift seen in the combined population (8.44, a 62% increase over the isolate mean).

Figure 4 shows the complementary *t*_f_ vs Δ*t* comparison plots that assess whether variation in *t*_f_ was primarily attributed to variations in Δ*t*, rather than to differences in *t*_i_. In *S. aureus*, slopes remained near unity in PBS (0.92–1.01) and TS (1.00–1.05), with small intercepts of 0.90–4.50 min and 1.61–7.95 min, respectively (Table S1). In *E. coli*, the slopes were less proportional (1.39–2.13 in PBS and 1.00–1.36 in LB), and the intercepts were larger (21.17–68.79 min in PBS and 25.46–141.40 min in LB), indicating that final timing retained a stronger onset-related component, especially in the combined LB population. Isolate-to-isolate variability was substantial. *E. coli* PBS intercepts ranged from 21.17 to 68.79 min among isolates 1–3 (mean 41.87 min), while the combined population (23.26 min) fell within that isolate range rather than outside it. In *E. coli* LB, isolates 1–3 ranged from 25.46 to 55.27 min (mean 40.48 min, a range equal to 74% of the mean), and in this instance the combined-population intercept (141.40 min) exceeded the isolate range by a wide margin. For instance, this is one comparison in this dataset where pooling colonies appear to introduce more variability than is present among the isolates themselves, a point we return to in Section 2.5. Same species, same media isolate comparisons therefore show that a substantial fraction of the variability otherwise attributed to population mixing is already present among individually isolated founder lineages, even though the two sources of variability are not always of equal magnitude.

**Figure 4:**
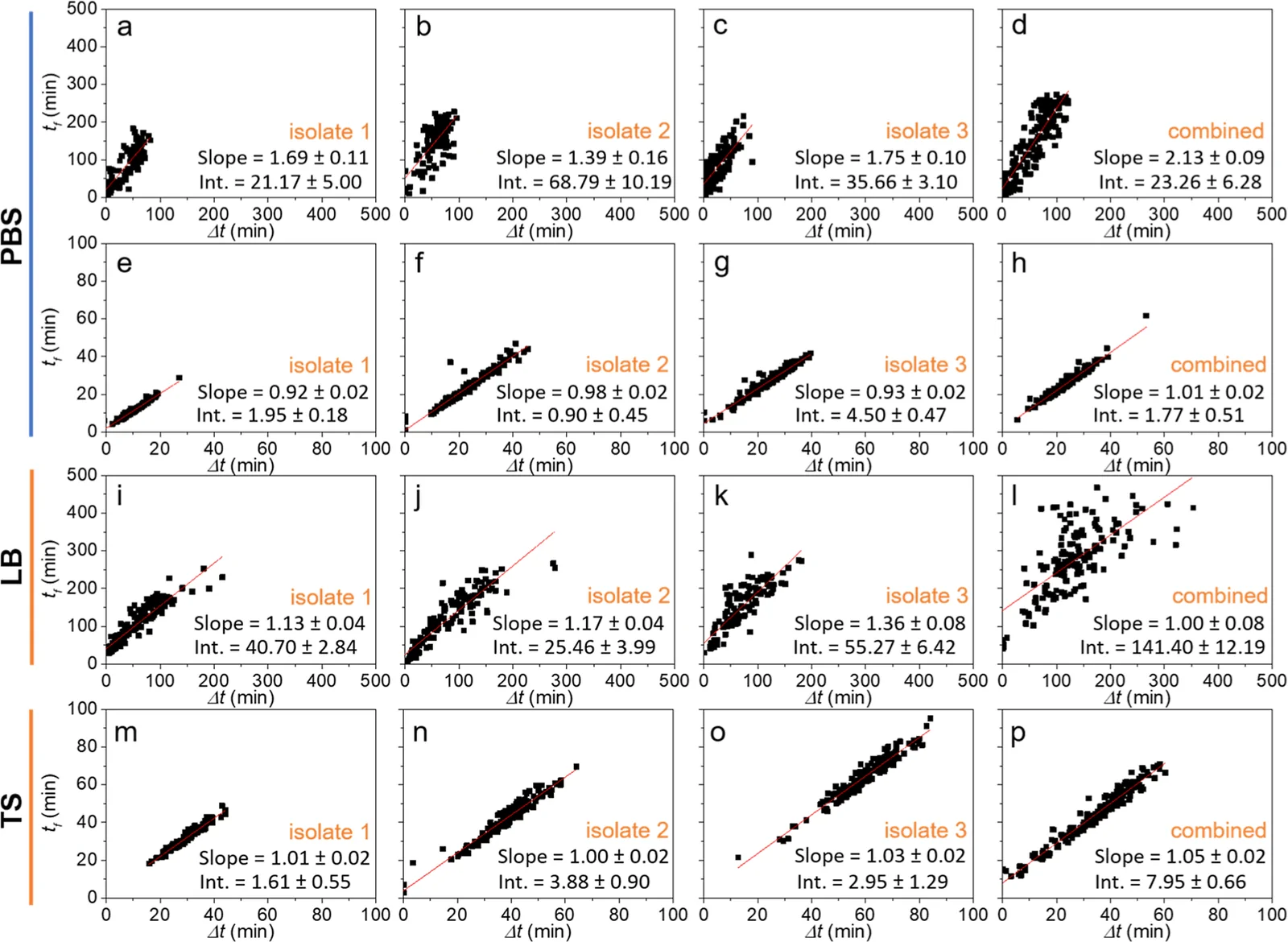
Relationship between final PI-positive transition time and transition duration across isolate-derived and combined populations. Scatter plots show the relationship between *t*_f_ and Δ*t* = *t*_f_ – *t*_i_ for individual bacterial cells during aPDT. Panels (a–d) correspond to *E. coli* in PBS, panels (e–h) to *S. aureus* in PBS, panels (i–l) to *E. coli* in LB, and panels (m–p) to *S. aureus* in TS. Fitted lines summarize timing alignment within each population. These plots were interpreted descriptively because *t*_f_ and Δ*t* are algebraically coupled. The power of excitation light was 0.66 ± 0.02 mW. Light dose varied between 4.75 ± 0.14 and 14.3 ± 0.4 J/cm^2^.

The relationship between *t*_i_ and Δ*t* was also examined to determine whether cells with delayed PI-entry onset also required longer times to complete the PI-positive transition (Figure S8). In *E. coli*, the distributions were broader along both axes, especially in PBS and in the combined LB population, showing that cells differed in both the timing of PI-entry onset and the duration of the transition. However, the points did not follow a common trend, indicating that delayed onset was not always accompanied by a longer transition duration. In *S. aureus*, *t*_i_ remained highly compressed in both PBS and TS, with most cells beginning PI uptake within a narrow time window, whereas variability was expressed mainly through differences in Δ*t*. This pattern supports the interpretation that PI-entry onset and post-onset progression represent partially separable components of the photodynamic response, with *E. coli* showing variability in both phases and *S. aureus* showing variability mainly after PI entry has begun.

### 2.4 Post-Treatment Regrowth Reveals Species-Dependent Recovery

To complement the single-cell microscopy analysis, bulk regrowth assays were performed using *E. coli* and *S. aureus* populations exposed to green light in the presence of the same Br_2_B concentration. *E. coli* populations were irradiated for 1 h, corresponding to a light dose of 7.74 ± 0.4 J/cm^2^, whereas *S. aureus* populations were irradiated for 30 min, corresponding to a light dose of 3.87 ± 0.4 J/cm^2^. These species-specific irradiation conditions were selected to produce sublethal photodynamic stress that allowed measurable post-treatment regrowth within the 15 h monitoring window for each organism. Therefore, the regrowth assay was designed to compare population-dependent recovery patterns within each species and should not be interpreted as a direct equal-dose comparison of photodynamic susceptibility between *E. coli* and *S. aureus*. After treatment, regrowth was monitored by measuring OD_600_ every 5 min for 15 h. The complete OD_600_ traces for all isolates-derived and combined populations are shown in Figure S9. The growth curves were then fitted using the modified Gompertz model (see Supporting Information), allowing extraction of the lag phase (λ) and maximum growth rate (μ_max_) (Figures S10 and S11 and Table S2), which were used to compare post-treatment recovery kinetics among populations.

The relationship between these two parameters is displayed in Figure 5a with representative regrowth traces shown in Figure 5b,c. The figure separates the two species into distinct recovery profiles. The *E. coli* isolate-derived populations formed a compact group with short lag phases (λ = 1.85–2.78 h) and relatively low maximum growth rates (μ_max_ = 0.082–0.093 OD_600_/h). In contrast, the *S. aureus* isolate-derived populations showed substantially longer λ (λ = 5.45–7.70 h) but higher maximum growth rates (μ_max_ = 0.104–0.140 OD_600_/h). Thus, under the applied treatment conditions, *S. aureus* populations required a longer recovery period before measurable regrowth but increased more rapidly once growth resumed. Nevertheless, the combined populations did not fall within the main isolate clusters. The combined *E. coli* population had the shortest λ among treated samples (λ = 1.07 h) but also the lowest maximum growth rate (μ_max_ = 0.078 OD_600_/h). Similarly, the combined *S. aureus* population showed a shorter lag phase (λ = 3.86 h) and lower growth μ_max_ (μ_max_ = 0.103 OD_600_/h) than most isolate-derived populations. As shown in Figure 5b,c, these positions indicate that the combined populations produced earlier detectable bulk growth but did not show faster subsequent growth. This pattern is consistent with early-recovering cells contributing disproportionately to the initial increase in OD_600_.^31^ Furthermore, isolate 8 presented a shorter lag phase and lower μ_max_ than the other isolates. In contrast, isolate 5 showed the longest λ (λ = 7.70 h) and the highest fitted growth rate (μ_max_ = 0.140 OD_600_/h). These differences indicate that independently derived colony populations can vary in both recovery delay and growth rate after treatment.

**Figure 5:**
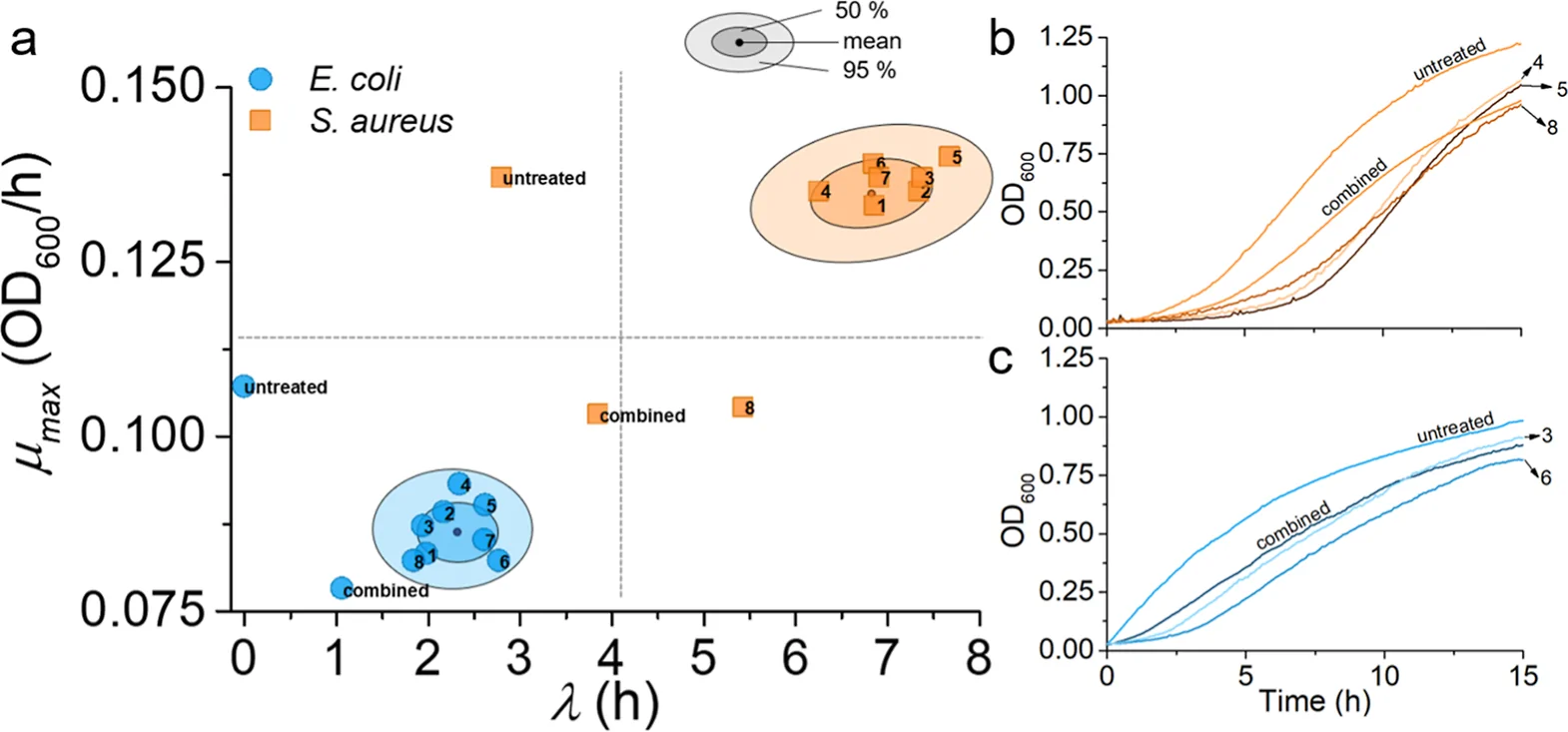
Post-aPDT regrowth heterogeneity. (a) Relationship between λ and μ_max_ during post-aPDT regrowth. Each point represents a single-colony-derived isolated population (numbered points), a mixed-founder combined population, or an untreated control fitted with the three-parameter modified Gompertz model. Blue circles indicate *E. coli* populations and orange squares indicate *S. aureus* populations. (b,c) Representative OD_600_ regrowth curves for selected *S. aureus* and *E. coli* populations, respectively, showing differences in recovery timing among isolate-derived, combined, and untreated populations. *E. coli* and *S. aureus* were treated using species-specific sublethal irradiation conditions selected to permit measurable regrowth during the 15 h monitoring period.

The variation in Gompertz lag phase among independently derived populations is consistent with the single-cell finding that photodynamic responses are heterogeneous rather than synchronous. However, the bulk lag parameter and the single-cell PI-entry parameters quantify different stages of the response: λ reflects the time required for surviving cells to generate measurable population growth after treatment, whereas *t*_i_, Δ*t*, and *t*_f_ describe the kinetics of PI entry during illumination. Therefore, the regrowth data support the general conclusion that recovery is species- and population-dependent, but they do not establish a direct correspondence between any individual PI-entry trajectory and subsequent outgrowth behavior. In particular, the shorter λ of the combined populations is consistent with earlier-recovering survivors contributing disproportionately to the bulk OD signal, rather than demonstrating that slower-recovering subpopulations were absent.

Individual isolates already showed substantial variability. This raised an important question: was this variability driven mainly by mutations accumulated during colony growth, or by non-genetic physiological differences among cells and founder lineages? We addressed this question in the following section.

### 2.5 Estimated Mutational Heterogeneity in Single-Colony and Mixed-Founder Inocula

Population structure adds context for interpreting single-cell PI-entry heterogeneity. Populations prepared from isolated colonies were treated here as single-colony-derived founder-lineage populations rather than as perfectly genetically uniform cultures. This is because a visible colony expands through many rounds of replication from one founder cell, and spontaneous replication errors that escape proofreading and mismatch repair can generate low-frequency mutant sublineages during colony growth.^12,13^ In contrast, the combined population contains cells from multiple colonies. This increases the number of founder lineages represented in the sample. The fraction of mutant-bearing cells may remain similar to that of a single colony, but the combined population contains more total cells with independent colony histories. How much do these genetic variables contribute to survival heterogeneity during aPDT?

To estimate this contribution, published mutation rates were converted to genome-level fixed mutation rates for both strains. For *E. coli*, the genome target was approximated as 5.36 Mb (chromosome plus two plasmids)^11^ and a central mutation-rate estimate of 2.20 × 10^–10^ mutations per nucleotide per generation gives a fixed mutation rate of 1.18 × 10^–3^ mutations per genome per generation, or about one fixed mutation every 850 replication cycles.^12,13^ For *S. aureus*, a 2.83 Mb and a rate of 2.70 × 10^–10^ mutations per nucleotide per generation give 7.64 × 10^–4^ mutations per genome per generation, or about one fixed mutation every 1300 replication cycles.^14,32^ (see Supporting Information for detailed calculations).

Applied to an overnight colony of approximately 3.3 × 10^9^ cells (∼31.6 generations from a single founder),^33^ these rate predict approximately 3.90 × 10^6^ mutations events for *E. coli* and 2.52 × 10^6^ for *S. aureus* during colony growth.^11–14,32,33^ Because mutations arising early in colony growth are inherited by many descendants, the resulting number of mutant-bearing cells exceeds the number of independent mutation events: approximately 1.2 × 10^8^ *E. coli* cells (∼3.7% of the colony) and 7.8 × 10^7^ *S. aureus* cells (∼2.4% of the colony) are expected to carry at least one newly acquired mutation relative to the founder. These percentages should not be read as the fraction of cells with a measurable phenotype, since most mutations are silent, neutral, deleterious, or unrelated to the PI-entry response measured here; rather, they establish mutation-derived heterogeneity as a plausible, low-level background feature of any colony-derived inoculum. This single-colony estimate reflects diversity within one founder lineage; mixing *k* equally contributing colonies instead combines *k* independent founder lineages, scaling the absolute number of mutation events and mutant-bearing cells approximately *k*-fold while leaving the percentage near ∼3.7% (*E. coli*) and ∼2.4% (*S. aureus*). Mixing 100 colonies, for example, would contribute approximately 1.2 × 10^10^ and 7.8 × 10^9^ mutant-bearing cells, respectively, while increasing founder-lineage diversity 100-fold. The key distinction is that single-colony expansion generates many mutant sublineages within one family tree, whereas mixed-founder sampling combines many independent family trees, raising the chance that rare genetic or physiological variants are represented without increasing the per-cell mutation rate.

A further filter is whether a mutated locus is both expressed under assay conditions and capable of altering the measured phenotype. Assuming that only 1–5% of genome-wide mutations fall in genes that could plausibly affect the cell wall,^21,22^ an *f*_env_ denote the fraction of loci expressed (0.01–0.05), the expressed, phenotype-relevant subset can be approximated as $P_{env}=1-e^{-\mu_{genome}gf_{env}}$ ; where *g* is the number of generations from founder to final population and μ_genome_ is the genome-wide mutation rate per generation (1.2 × 10^–3^ for *E. coli* and 7.7 × 10^–4^ for *S. aureus*). For a colony containing 3.3 × 10^9^ cells (*g* = 31.6 generations), this gives an estimated probability of at least one cell–wall associated mutation of 0.038–0.190% for *E. coli* and 0.024–0.120% for *S. aureus* (see Supporting Information section for derivation). These probabilities correspond to approximately 1.3 × 10^6^ to 6.3 × 10^6^ *E. coli* cells and 7.9 × 10^5^ to 4.0 × 10^6^ *S. aureus* cells whose lineages are expected to carry at least one mutation with the potential to affect cell–wall composition.

These predictions can be tested against the isolate-to-isolate and isolate-to-combined differences in *t*_f_ range within each species–medium combination, holding envelope biology and illumination constant. In *E. coli* PBS, isolates 1–3 spanned a *t*_f_ range of 180.8–218.5 min (19% of the 203.6 min isolate mean), and the shift to the combined population (268.0 min) was a further 32%, both far exceeding what the ∼3.7% predicted mutant fraction could produce in a whole-population statistic. The gap is wider still in LB, where isolates 1–3 spanned only 16% of their mean (223.2–261.6 min, mean 248.1 min) yet the combined population (425.5 min) shifted by 72%, indicating that combining colonies introduced more variability than existed among the isolates themselves. This pattern is more consistent with mixed-founder physiological diversity than with the mutation model. *S. aureus* comparisons were wider overall: in PBS, isolates 1–3 spanned 24.5–46.0 min (59% of the 36.2 min mean), comparable to the 53% isolate-to-combined shift (55.2 min); in TS, isolates 1–3 spanned 40.5–73.7 min (55% of the mean), while the combined population (60.5 min) landed almost exactly on the isolate mean (60.4 min, a 0.3% shift). It can be noticed here that the isolate-to-isolate variability matched or exceeded the isolate-to-combined shift in three of the four species–medium combinations, and in every condition both exceeded what a 2–4% mutant-bearing fraction could plausibly explain. The exception, *E. coli* in LB, suggests that population mixing can introduce physiological variability beyond that captured among individual isolates.

This comparison helps to calibrate how much of the observed single-cell aPDT heterogeneity should be attributed to mutation. Mutation-derived heterogeneity likely contributes to rare outlier trajectories, founder effects, or colony-to-colony differences, particularly for mutations affecting envelope permeability, LPS/capsule/porin composition in *E. coli*, or peptidoglycan and teichoic-acid-related properties in *S. aureus*.^21,22^ However, founder-lineage variability within a single species and medium is, in most conditions, already comparable to or larger than the variability attributable to mutation accumulation, so the observed PI-entry timing differences are more consistent with physiological heterogeneity (PS access, nutrient-dependent repair capacity, oxidative-stress state, and envelope architecture) as the dominant drivers. The *E. coli* LB exception indicates that population mixing can, in some cases, draw on a broader range of physiological states rather than simply adding mutations. Mutation-derived diversity is therefore best understood as a low-frequency background contributor that coexists with, but does not replace, non-genetic phenotypic heterogeneity among founder lineages.

### 2.6 Mechanistic Interpretation: Envelope Access, Nutrient Context, and Physiological State

The species comparison is consistent with known differences in PS access across bacterial envelopes. aPDT susceptibility depends not only on ^1^O_2_ yield but also on PS localization, cell-envelope permeability, antioxidant capacity, efflux, and physiological state.^7,8,23,34^ Gram-negative bacteria are more difficult to photoinactivate because the outer membrane restricts the access of many PSs, whereas Gram-positive bacteria possess a simpler, porous peptidoglycan envelope. Uptake assays were consistent with this difference in envelope architecture. In PBS, *E. coli* accumulated approximately 12% of the applied Br_2_B, whereas *S. aureus* accumulated approximately 33% (Figure 6). A similar trend was observed in nutrient-rich media, although uptake decreased for both species. In LB, *E. coli* accumulated approximately 8% of Br_2_B and in TS, *S. aureus* accumulated approximately 26% of Br_2_B. Thus, nutrient-rich media reduced the apparent association of Br_2_B with bacterial cells, likely because medium components partially retained or interacted with the PS. These uptake differences are consistent with the direct single-cell distributions. *S. aureus* showed shorter PI-uptake durations, whereas *E. coli* showed longer and more broadly distributed Δ*t* values. The *t*_f_ based regressions provide additional descriptive support, but they were not treated as independent mechanistic tests because *t*_f_ is calculated from *t*_i_ and Δ*t*. In *S. aureus*, final PI fluorescence timing was closely associated with the duration of PI uptake after onset. In *E. coli*, final PI fluorescence timing reflected both delayed PI-uptake onset and the duration of PI uptake, especially under LB conditions. In addition, nutrient-rich media can provide metabolic resources that support repair, protein turnover, redox recovery, and membrane remodeling after photodynamic damage. However, peptides, proteins, and amino acids in rich media can also react with ^1^O_2_ and radicals reducing the effective photodynamic dose.^35–37^ Therefore, differences between PBS and nutrient-rich media likely reflect both bacterial physiological changes and chemical changes in the photodynamic microenvironment.

**Figure 6:**
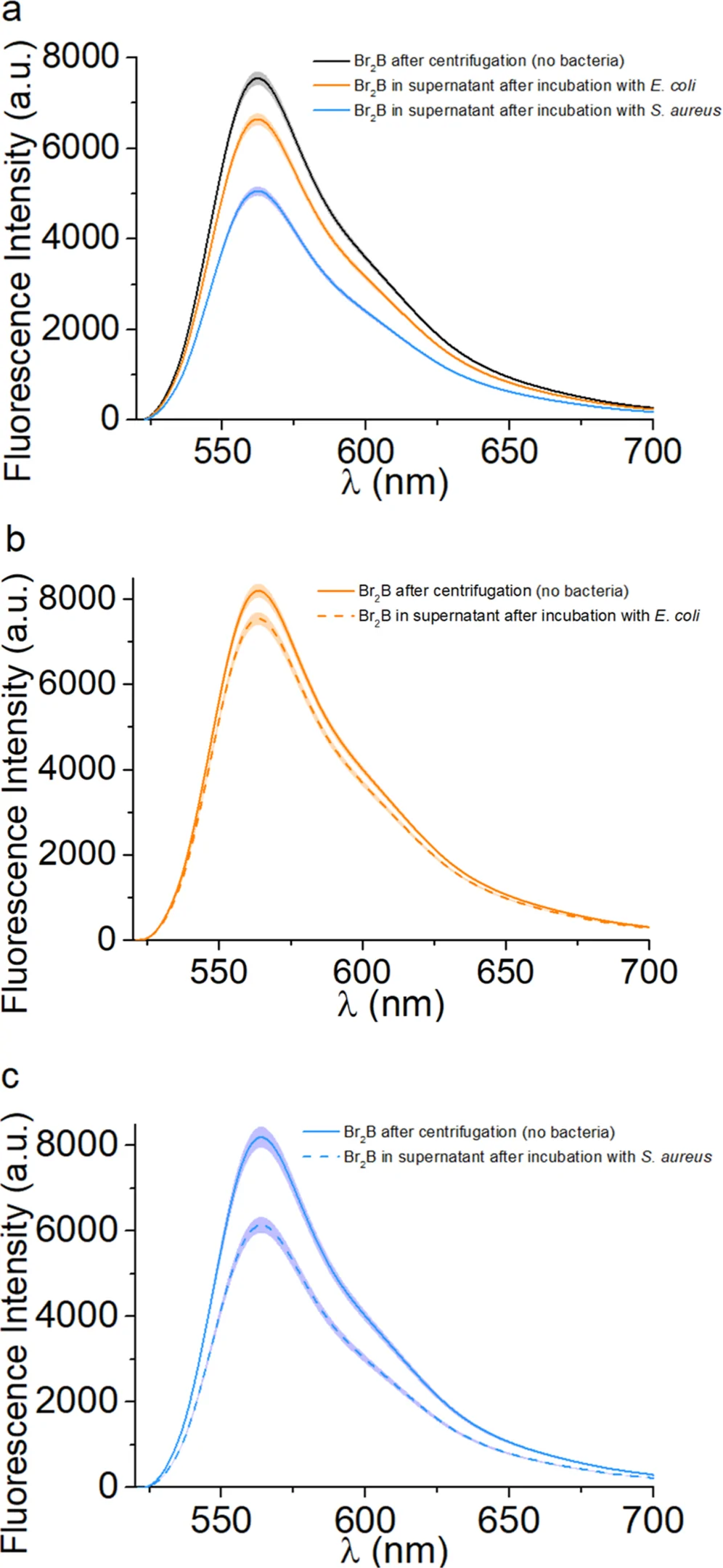
Br_2_B uptake by *E. coli* and *S. aureus* estimated by supernatant fluorescence depletion. Fluorescence emission spectra of Br_2_B were recorded after centrifugation in the absence of bacteria and compared with the fluorescence remaining in the supernatant after incubation with bacteria. (a) Br_2_B uptake in PBS showed greater fluorescence depletion after incubation with *S. aureus* than with *E. coli*, corresponding to approximately 33% and 12% uptake, respectively. (b) In LB, *E. coli* showed lower apparent Br_2_B uptake, with approximately 8% depletion relative to the control. (c) In TS, *S. aureus* showed approximately 26% Br_2_B uptake relative to the control. The decrease in supernatant fluorescence indicates association of Br_2_B with bacterial cells, while medium-matched controls account for changes in Br_2_B emission caused by nutrient-rich media.

Furthermore, differences among species in their protection against ^1^O_2_ and downstream oxidative stress are also worth considering. *E. coli* relies primarily on indirect protection through its outer membrane, stress regulons such as OxyR, SoxRS, and RpoS, redox-recovery pathways, and repair systems.^3,6,35,38^ *S. aureus* produces the membrane-associated carotenoid staphyloxanthin, which contributes to oxidative-stress tolerance and virulence by protecting against oxidant killing.^39,40^ *S. aureus* and *E. coli* also use catalase, superoxide dismutases, alkyl hydroperoxide reductases, thiol-redox systems, and methionine sulfoxide reductases to survive oxidative damage. These systems may not directly detoxify ^1^O_2_, but they can mitigate secondary oxidative stress and repair downstream damage after aPDT.

These data supports a model in which single-cell aPDT response is shaped by the interaction of PS access, envelope architecture, nutrient-dependent repair capacity, oxidative-stress state, and background population structure. Genetic heterogeneity is expected even in colony-derived populations, but the dominant timing patterns observed here are more consistent with phenotypic and physiological heterogeneity than with mutation accumulation alone.

### 2.7 Limitations

Several limitations should be considered. First, PI fluorescence reports envelope permeabilization and nucleic-acid binding rather than instantaneous loss of viability. PI can overestimate or misrepresent viability in some bacterial contexts, including adherent cells, cells with altered membrane potential, or samples containing extracellular nucleic acids.^24,25^ Therefore, *t*_i_, *t*_f_, and Δ*t* should be interpreted as PI-entry kinetics rather than direct death times. Second, Br_2_B uptake was measured in planktonic media. These measurements do not determine how much Br_2_B was associated with each individual cell. Single-cell Br_2_B fluorescence measurements acquired before illumination showed similar emission intensities before the addition of PI. However, the signal was too weak to support a reliable comparison of Br_2_B uptake among cells.

Finally, the mutational heterogeneity estimates are order-of-magnitude calculations, not direct sequencing measurements of the specific bacterial strains used in this study. These estimates assume neutral mutation accumulation, no strong selection during colony growth, and simplified genome targets. Plasmid copy number, colony age, medium composition, incubation time, stress-induced mutagenesis, and population size can alter the total number of replicated sites and generations.^41–44^ Moreover, the presence of a mutation does not imply a detectable phenotype; many mutations are silent, neutral, deleterious, or unrelated to envelope permeability and oxidative-stress response.

## 3 Conclusion

This study shows that aPDT produces species-specific single-cell response dynamics in *E. coli* and *S. aureus*. Time-resolved PI fluorescence microscopy provided insights on envelope permeabilization in individual bacteria and separated the response into *t*_i_, *t*_f_, Δ*t*. In *E. coli*, heterogeneity was distributed across both delayed *t*_i_ and prolonged Δ*t*, particularly under nutrient-rich LB conditions. In contrast, *S. aureus* showed a more compressed *t*_i_, with variability expressed mainly after PI entry had begun. This difference was consistent with the higher Br_2_B uptake measured in *S. aureus* and with the different envelope architectures of Gram-positive and Gram-negative bacteria. Post-treatment regrowth assays further showed that recovery after aPDT differed among founder-lineage populations, with *E. coli* generally showing shorter λ and lower μ, whereas *S. aureus* showed longer recovery delays followed by faster growth once regrowth began. These bulk recovery profiles represent a later stage of the response than single-cell PI-entry kinetics, but together they reinforce the conclusion that aPDT response is heterogeneous, species-dependent, and shaped by both envelope access and physiological state. Mutation-rate estimates provide an additional framework for interpreting these results. Single-colony-derived cells should be considered highly related founder-lineage populations rather than perfectly uniform isogenic populations, while combined samples represent mixed-founder populations containing multiple independent lineage histories. However, mutation-derived diversity is expected to act primarily as a low-frequency background contributor rather than as the dominant driver of the observed timing patterns. The data are more consistent with heterogeneity arising from PS uptake, envelope structure, nutrient context, repair capacity, and cell-to-cell physiological variation. Future work will focus on determining if cells with low PS uptake are the same cells that show delayed PI entry or recover after treatment. Furthermore, additional studies using other PSs, biofilm-like samples, and infection-relevant media will help determine how broadly these response patterns apply.

## 4 Materials and Methods

### 4.1 Materials

Chemical reagents, Lennox Broth (LB), Tryptic Soy (TS) broth, solvents, PI, and Phosphate Buffer Saline (PBS) were purchased from MilliporeSigma. Coverslips 22 × 22 mm were purchased from Karter Scientific Labware Manufacturing. Consumables for bioassays were purchased from Fisher Scientific. PLA filament was purchased from Amazon. Br_2_B was prepared as previously described.^5^

### 4.2 Instrumentation

The sample holders to make the agarose pads were printed using a Prusa i3MK3S 3D printer purchased from Prusa Research (Prague, Czech Republic). Micrographs were acquired with a Nikon ECLIPSE Ti2 Inverted Fluorescence Microscope coupled to a four-channel LED light engine (Lumencor SPECTRA, Beaverton, OR, USA). A temperature-regulated monochrome CCD camera with a Sony ICX285AL EXview HAD sensor and a quantum efficiency of 65% was used to acquire the images. For bulk experiments OD at 600 nm was recorded using a Multi-Mode Microplate Reader (Agilent BioTek Synergy HTX). Post aPDT treatment was performed irradiating a Corning Costa 24-well clear microplates with lid with a custom-build LED panel emitting green light with a power of 2.15 ± 0.3 mW.

### 4.3 Imaging Platform

Single-cell aPDT experiments were performed using a custom 3D-printed microscopy holder designed to immobilize bacteria using an agarose pad. The holder has two sections: a lower stage adapter and an upper sample-retaining chamber. The lower stage adapter is a bottom rectangular that fits onto the microscope stage holder. It provides mechanical support and contains the central opening that allows the 60× objective to access the coverslip from below. The upper sample-retaining chamber is the top section that holds the agarose pad and bacterial sample in position. It contains the circular sample well and lateral arms/screw holes used to align or secure the piece onto the lower base. Both parts were designed and modeled using Autodesk Fusion 360 (Autodesk Inc., USA). The printing parameters were adjusted using PrusaSlicer-2.6.1 (Prusa Research, Czech Republic). The layer height was 0.15 mm, vertical shells of 4 lines (1.6 mm), horizontal shells of 4 lines (0.6 mm), and 50% infill. A 0.4 mm nozzle was used to print the holders. The printing temperatures were 215 °C for the extruder and 60 °C for the bed. The device consisted of a central imaging window that aligned the sample with the microscope objective and lateral supports that secured the coverslip/agarose assembly in a fixed position. Before use, the holder and coverslips were washed with 70% ethanol, dried, and assembled.

### 4.4 Sample Preparation for Microscopy

Coverslips (22 × 22 mm) were submerged in ethanol and sonicated for 30 min. The ethanol was then removed and replaced with acetone, and sonication was repeated for an additional 30 min. After removal from the acetone, residual solvent was evaporated from the coverslip surface under a stream of nitrogen gas. A coverslip was placed in the aforementioned lower stage adapter (Figure 1). A 1% agarose solution was prepared in a falcon tube and dissolved in PBS or the corresponding broth (LB or TS) by heating it to near boiling, about 85 °C, until the solution becomes completely clear. Followed by addition of PI and Br_2_B for the aPDT experiments or PI only for control experiments. The agarose solution was poured into the upper-sample retaining chamber and allowed to cool down to room temperature. The chamber was flipped over and 2.5 μL of a bacterial suspension was poured on top. The chamber was flipped back to its original position and fitted into the lower stage adapter. The final concentration of PI and Br_2_B in the agarose pad was 5 μM. PI was dissolved in PBS buffer, and Br_2_B was dissolved in acetonitrile (ACN). The content of ACN in the pad was kept below 2% to avoid cytotoxicity by the solvent. For each species, medium, and population condition, 150 individual cells were analyzed corresponding to 600 single-cell trajectories per species and medium condition and 2400 total single-cell trajectories across the full microscopy dataset.

### 4.5 Brightfield and Fluorescence Microscopy

Time-lapse brightfield and fluorescence microscopy was performed using a Nikon Eclipse Ti2 inverted microscope equipped with a four-channel LED light engine (Lumencor SPECTRA, Beaverton, OR, USA). PI fluorescence was monitored using green excitation light centered at 542/33 nm. The excitation light was further spectrally filtered using a 550/20 nm excitation filter and directed to the sample through a 585 nm cut-on dichroic mirror. Fluorescence emission was collected through a 630/40 nm emission filter. The same green illumination was used to excite Br_2_B and induce photodynamic oxidative stress. Because Br_2_B has a relatively low fluorescence quantum yield (Φ_F_ = 0.14),^5^ PI fluorescence could be monitored using exposure times and camera-gain settings selected to maximize the red PI signal while limiting interference from Br_2_B emission. Images were acquired with a Nikon Plan Apochromat 60× oil-immersion objective (NA = 1.4). Fluorescence emitted by the samples was collected through the same objective and recorded using a temperature-regulated monochrome CCD camera equipped with a Sony ICX285AL EXview HAD sensor (quantum efficiency: 65%). Brightfield transmission images were acquired using the same objective. The green-light irradiance measured at the objective output was 0.66 ± 0.02 mW/cm^2^. Time-lapse images of *E. coli* were acquired every 2 min for up to 180 frames, corresponding to a maximum irradiation time of 360 min and a light dose of 14.3 ± 0.4 J/cm^2^. *S. aureus* images were acquired every 1 min for 2 h, corresponding to a light dose of 4.75 ± 0.14 J/cm^2^. Experiments were performed at room temperature and continued until cells reached their maximum fluorescence intensity in the red channel. For control experiments used to assess the absence of bacterial growth within irradiated regions, images were acquired using both the 60× oil-immersion objective and a Nikon Plan Apochromat 10× air objective. One independent time-lapse field of view was analyzed for each population because the same field had to be tracked throughout the prolonged illumination experiment.

## Supplementary Material



## Notes

Materials and experimental methods; representative brightfield images showing the region exposed to green-light irradiation; brightfield and PI fluorescence images of *E. coli* at the boundary of the irradiated region; light-only and dark controls for *E. coli* and *S. aureus*; a schematic representation of isolated colonies and combined bacterial populations; a summary table of single-cell kinetic parameters extracted from PI fluorescence trajectories during aPDT; plots showing the relationship between *t*_i_ and Δ*t* across isolate-derived and combined populations; post-aPDT regrowth curves for isolate-derived and mixed-founder bacterial populations; analysis of bacterial growth curves using the modified Gompertz equation; modified Gompertz fits for post-aPDT regrowth of *E. coli* and *S. aureus* populations; Gompertz parameters extracted from post-aPDT bulk regrowth curves after baseline correction; calculations used to estimate mutational heterogeneity in single-colony and mixed-colony populations; and supporting references (PDF)
