# Supplementary material for: Single-Cell Membrane-Permeabilization Kinetics Reveal Species-Specific Responses to Antimicrobial Photodynamic Treatment
Source: ACS Omega. 2026 Sep 15;11(38):58330–42. doi: 10.1021/acsomega.6c09003 (PMC13628624; doi:10.1021/acsomega.6c09003)
Supplement: Supplementary file 1 [file ao6c09003_si_001.pdf]

Supporting Information

Single-Cell Membrane-Permeabilization Kinetics Reveal Species-Specific Responses to Antimicrobial Photodynamic Treatment

*Omnia Ahmed[1], Sol R. Martínez[2], Taufiq Khan[1], Nitika Paudyal[1], Yusra Amena[1], Mahmoud Abouelyazid[3], Andrés M. Durantini[1]**

[1]Department of Chemistry, Southern Illinois University Edwardsville, Edwardsville, Illinois 62026, United States.

[2]National Corn to Ethanol Research Center, Southern Illinois University Edwardsville, Edwardsville, Illinois 62025, United States.

[3] Department of Electrical and Computer Engineering, Texas A&M University, College Station, Texas 77843, United States.

*E-mail address: adurant@siue.edu

Table of Contents

SUPPORTING MATERIAL	Page
• **Materials and experimental methods**	S3
• **Figure S1.** Representative brightfield image of *E. coli* sample showing the region exposed to green-light irradiation	S5
• **Figure S2.** Brightfield and PI fluorescence imaging of *E. coli* at the boundary of the irradiated region	S6
• **Figure S3.** Control of *E. coli* in the absence of Br_2B after green-light irradiation	S6
• **Figure S4.** Dark control of *E. coli* in the presence of Br_2B	S7
• **Figure S5.** Control of *S. aureus* in the absence of Br_2B after green-light irradiation	S7
• **Figure S6.** Dark control of *S. aureus* in the presence of Br_2B	S8
• **Figure S7.** Schematic representation of isolated colonies and combined bacterial populations	S8
• **Table S1.** Summary of single-cell kinetic parameters extracted from PI fluorescence trajectories during aPDT	S9
• **Figure S8.** Relationship between t_i and Δt across isolate-derived and combined populations	S10
• **Figure S9.** Post-aPDT regrowth of isolate-derived and mixed-founder bacterial populations	S11
• **Analysis of bacterial growth curves using the modified Gompertz equation**	S11
• **Figure S10.** Modified Gompertz fits for post-aPDT regrowth of *E. coli* populations	S12
• **Figure S11.** Modified Gompertz fits for post-aPDT regrowth of *S. aureus* populations	S13
• **Table S2.** Modified Gompertz parameters extracted from post-aPDT bulk regrowth curves after baseline correction	S14
• **Calculation to estimate mutational heterogeneity in single-colony and mixed-colony populations**	S14
• References	S17

Materials and experimental methods

Strains, culture conditions, and colony selection. *E. coli* UMN026 and *S. aureus* SA113 (both ATCC) cultures were preserved in 10% glycerol (v/v) and 90% LB broth (v/v) and 10% glycerol (v/v) and 90% TS broth (v/v) at -80 °C, respectively. Then, *E. coli and S. aureus* were streaked onto LB agar and TS agar plates, respectively, and incubated aerobically at 37 °C for 18 h. The initial streak region showed confluent bacterial growth, followed by progressively diluted streaks that yielded isolated colonies. Cells were collected with an inoculating loop either from the confluent growth region, representing a mixed colonies (combined) population, or from individual well-isolated colonies, representing single-colony-derived populations. Each loopful was suspended in a microcentrifuge tube containing the appropriate medium for the experiment: PBS for nutrient-deficient conditions or LB broth for *E. coli* and TS broth for *S. aureus* under nutrient-rich conditions. Although all colonies originated from the same pure isolate stock, each colony-derived population was prepared from a different well-isolated colony collected from a freshly prepared agar plate. Because of the time required for sample preparation and long-term imaging, these populations were measured on separate days.

Image analysis and trajectory fitting. Images were processed in FIJI-ImageJ.[1] Stage drift was corrected with the GDSC plug-in (Utils® Align images),[2] using the final PI image as the reference frame. Cells in the imaging area were identified after thresholding with the Analyze Particles function. Particle-size ranges were set to 50-300 pixels for *E. coli* and 20-100 pixels for *S. aureus*. The mean PI intensity of each cell was measured over time and exported as an individual fluorescence trajectory. Because the number of cells varied among imaging regions, 150 cells were included in the final analysis for each experimental condition. The trajectories were fitted with a custom Python script using a four-parameter Boltzmann function (Figure 1f). Fitting started after the minimum-intensity frame and continued until 20 frames after the maximum-intensity frame. Trajectories were excluded when the fit did not converge or when the fluorescence signal did not reach a plateau, defined as a mean positive slope greater than 0.01 over the final 10 data points. The onset and final times of PI uptake were calculated using the tangent to the fitted curve at t_0. The intersection between this tangent and the fitted initial fluorescence level (I_i) was defined as t_i, whereas the intersection between the same tangent and the fitted final fluorescence level (I_f) was defined as t_f. The reported PI-uptake duration was calculated as $\Delta t = t_f - t_i$. Thus, Δt refers to the PI-uptake transition duration and not to the fitted Boltzmann width parameter. Frame numbers were converted to minutes according to the acquisition interval used for each experiment. Box plots and

scatter plots were prepared and analyzed using OriginPro (OriginLab Corporation, Northampton, MA, USA).

Microplate growth-recovery assays following photodynamic treatment. Post-treatment growth recovery was monitored in clear, lidded 24-well microplates by measuring optical density at 600 nm (OD_{600}). Each well contained a final volume of 1.0 mL. *S. aureus* SA113 cultures were prepared in TS broth, whereas *E. coli* UMN026 cultures were prepared in LB broth. Br_2B was added to a final concentration of 5 μM in all isolate-derived and mixed-colony bacterial cultures. For each strain, the plate included eight individual isolate or single colony-derived populations (isolates 1-8), a combined population, LB- or TS-only controls, bacteria-only controls (LB + *E. coli* and TS + *S. aureus*), and PS-only controls (LB or TS + Br_2B). (Figure S9). The *E. coli* plates were irradiated for 60 min with a green LED source centered at 520 nm and an irradiance of 2.15 ± 0.3 mW/cm^2, corresponding to a light dose of 7.74 ± 0.4 J/cm^2. *S. aureus* cultures were irradiated under the same illumination conditions for 30 min, corresponding to a light dose of 3.87 ± 0.4 J/cm^2. Different irradiation times were used because the goal of the assay was to establish species-specific sublethal treatment conditions that allowed measurable regrowth during the 15 h monitoring window. These conditions were not intended as an equal-dose comparison of photodynamic susceptibility between *E. coli* and *S. aureus*. Immediately after irradiation, plates were transferred to a microplate reader and incubated at 37 °C. OD_{600} was monitored every 5 min for 15 h. Orbital shaking was applied for 4 min during each 5-min cycle and stopped before OD_{600} acquisition to minimize motion-related optical artifacts.[3] The resulting growth curves were used to compare post-treatment recovery kinetics among isolate-derived and mixed-founder populations using the modified Gompertz equation (SI section 2).[4]

Br_2B uptake assay. Br_2B uptake by *E. coli* and *S. aureus* was quantified by fluorescence depletion from the extracellular medium. A single isolated colony of each strain was used to inoculate the corresponding nutrient-rich medium, LB broth for *E. coli* and TS broth for *S. aureus*. Cultures were incubated aerobically at 37 °C until they reached an OD600 of approximately 0.5. Cells were then collected by centrifugation and washed three times with PBS to remove residual medium components. The washed bacterial pellets were then resuspended to approximately 10^8 CFU/mL in the appropriate assay medium. For E. *coli*, Br_2B uptake was evaluated in PBS and LB. For *S. aureus*, Br_2B uptake was evaluated in PBS and TS. Br_2B was added to each bacterial suspension to a final concentration of 5 μM, and samples were incubated for 30 min at room temperature in the dark. After incubation, cells were pelleted by centrifugation at 8000 rpm for 5 min. The supernatant

was transferred to a quartz cuvette with a 10 mm path length, and fluorescence emission spectra were acquired using the same instrumental settings for all samples. A bacteria-free reference sample containing 5 μM Br_2B in PBS, LB, or TS was prepared and measured under the same conditions. For each condition, the fluorescence maximum of the reference spectrum was assigned as 100%. The decrease in the fluorescence maximum of the supernatant after incubation with bacteria was used to estimate the fraction of Br_2B associated with the bacterial cells. The percentage of Br_2B uptake by the bacterial strains in PBS was 12% for *E. coli* and 33% for *S. aureus*. These values decreased to 8% in LB for *E. coli* and 26% in TS for *S. aureus*.

Statistical analysis. For each species and experimental condition, three independently isolated colony-derived populations were analyzed as biological replicates (n = 3). Individual cells were analyzed as single-cell measurements within each colony-derived or combined population, whereas the separately prepared colony-derived populations were treated as the independent biological replicates. Cell-level kinetic parameters, including t_i, t_f, and Δt, were summarized separately for each colony-derived population before colony-level comparisons were performed. Data are presented as individual colony-derived populations together with the mean ± standard deviation (SD). Growth curves were fitted independently for each population using the modified Gompertz model to estimate λ, μ_{max}, and asymptotic growth yield (A). Relationships among kinetic parameters were evaluated by linear regression and reported with the fitted slope, intercept, coefficient of determination (R^2).

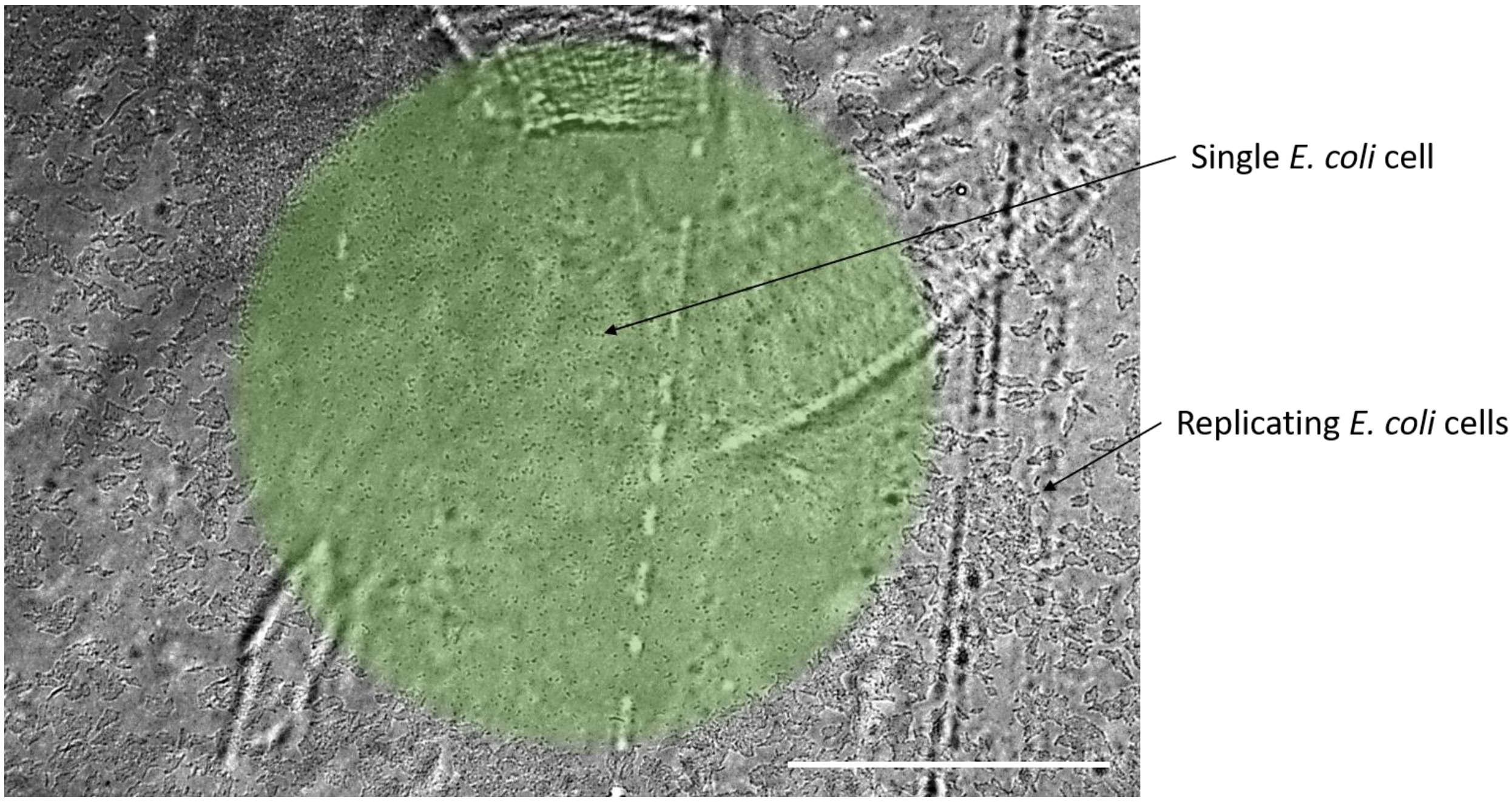

Figure S1. Representative brightfield image of *E. coli* sample showing the region exposed to green light irradiation. Bacterial cells located within the irradiated region appear inactivated, whereas cells outside the illuminated area continue to replicate and expand. The image was acquired after 3 h of treatment. Light dose was 7.1 ± 0.4 J/cm^2. Scale bar: 100 µm. Image was acquired using a 10× objective.

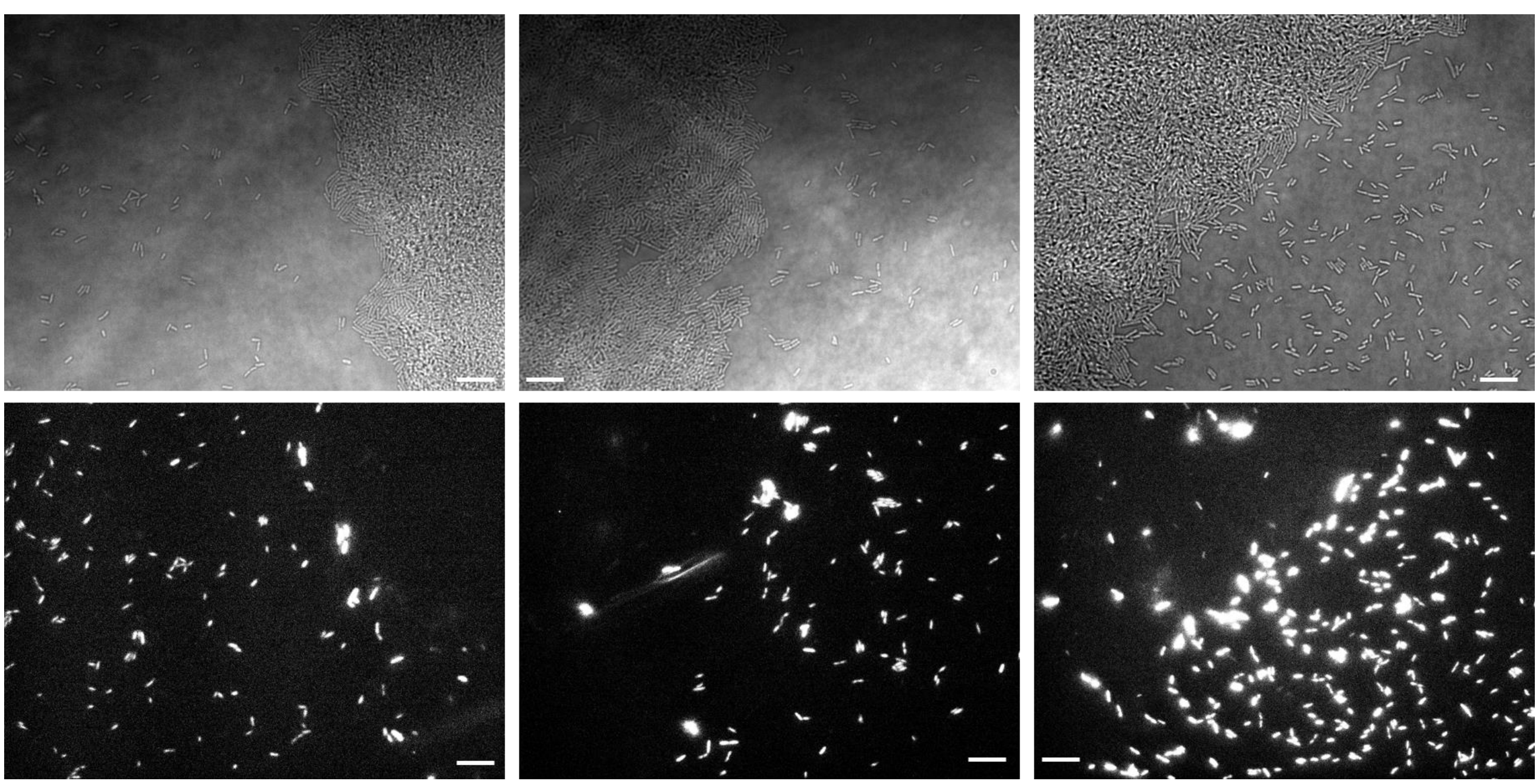

Figure S2. Brightfield and PI fluorescence imaging of *E. coli* at the boundary of the irradiated region. Representative matched brightfield images (top row) and corresponding PI fluorescence images (bottom row) acquired at 6 h after the green-light photodynamic treatment. The brightfield

images show a dense bacterial population outside the irradiated area and isolated cells within the treated region. Light dose was 14.3 ± 0.4 J/cm^2. Scale bars, 10 µm. Image was acquired using a 60× objective.

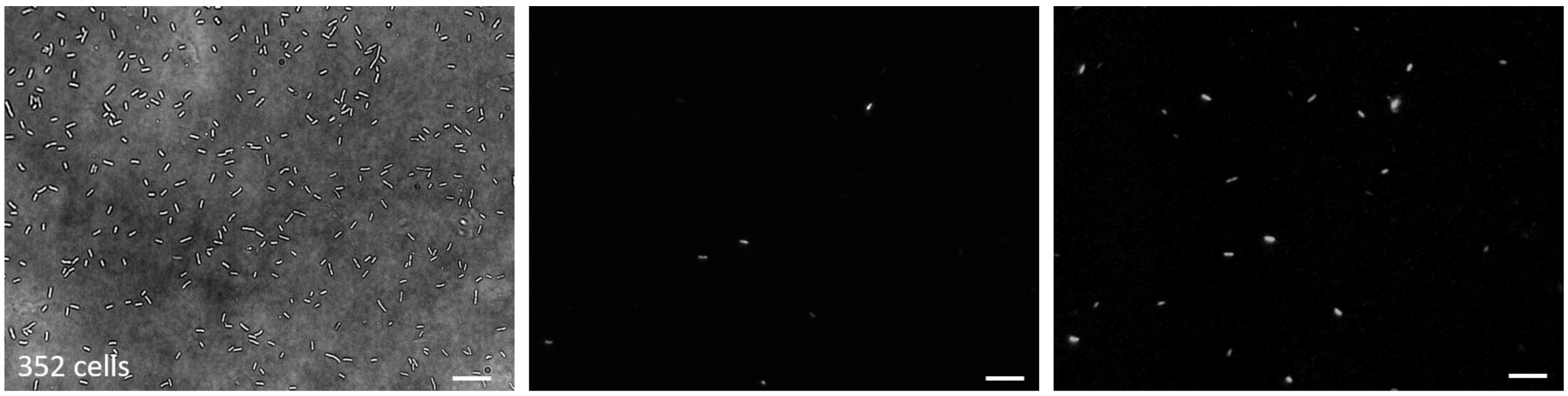

Figure S3. Control of *E. coli* in the absence of Br_2B after green-light irradiation. Brightfield image (left) of a representative field containing 352 bacterial cells, together with the corresponding PI fluorescence images acquired before irradiation (middle) and after 6 h of green-light exposure (right). The total light dose was 14.3 ± 0.4 J/cm^2. Agarose pad was prepared with PBS. Scale bars: 10 µm. Image was acquired using a 60× objective.

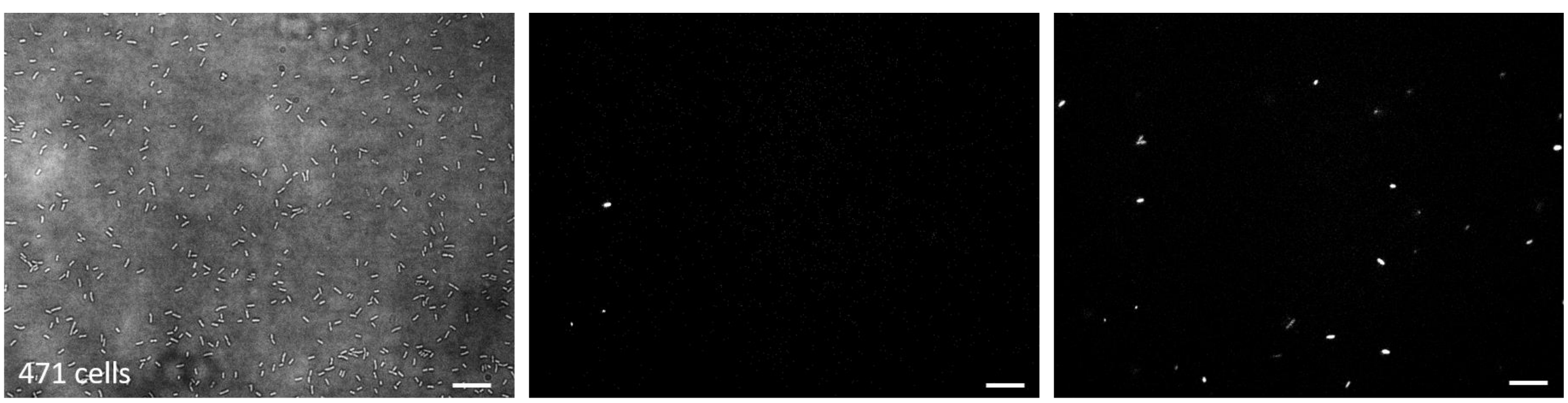

Figure S4. Dark control of *E. coli* in the presence of Br_2B. Brightfield image (left) of a representative field containing 471 bacterial cells, with corresponding PI fluorescence images acquired at the beginning (middle) and after 6 h of incubation (right). Agarose pad was prepared with PBS. Scale bars, 10 µm. Image was acquired using a 60× objective.

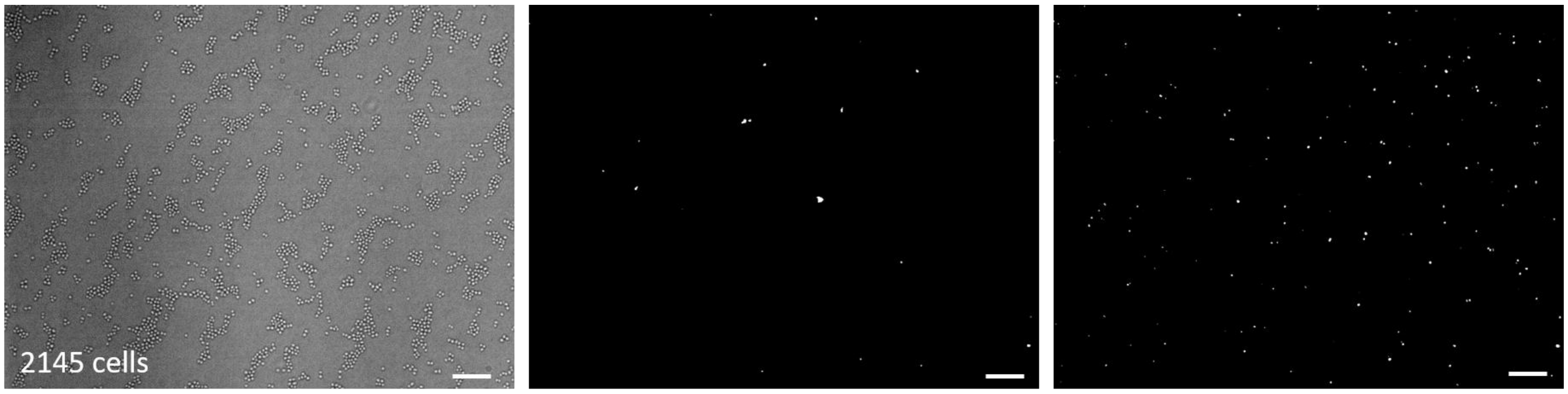

Figure S5. Control of *S. aureus* in the absence of Br_2B after green-light irradiation. Brightfield image (left) of a representative field containing 2145 bacterial cells, together with the corresponding PI fluorescence images acquired before irradiation (middle) and after 2 h of green-light exposure (right). The total light dose was 4.75 ± 0.14 J/cm^2. Agarose pad was prepared with PBS. Scale bars: 10 µm. Image was acquired using a 60× objective.

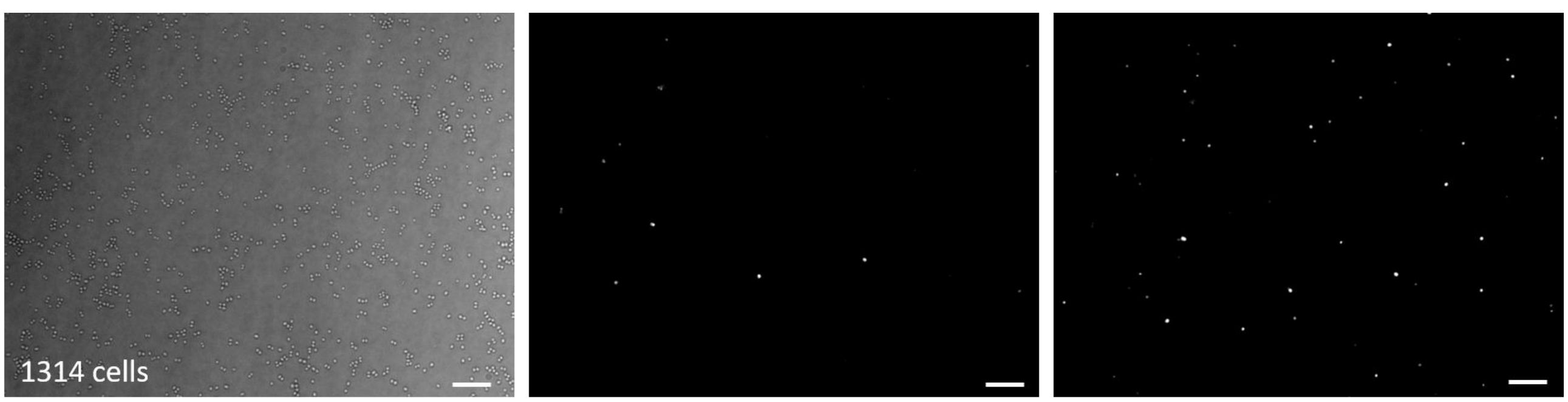

Figure S6. Dark control of *S. aureus* in the presence of Br_2B. Brightfield image (left) of a representative field containing 1314 bacterial cells, with corresponding PI fluorescence images acquired at the beginning (middle) and after 2 h of incubation. Agarose pad was prepared with PBS. Scale bars, 10 µm. Image was acquired using a 60× objective.

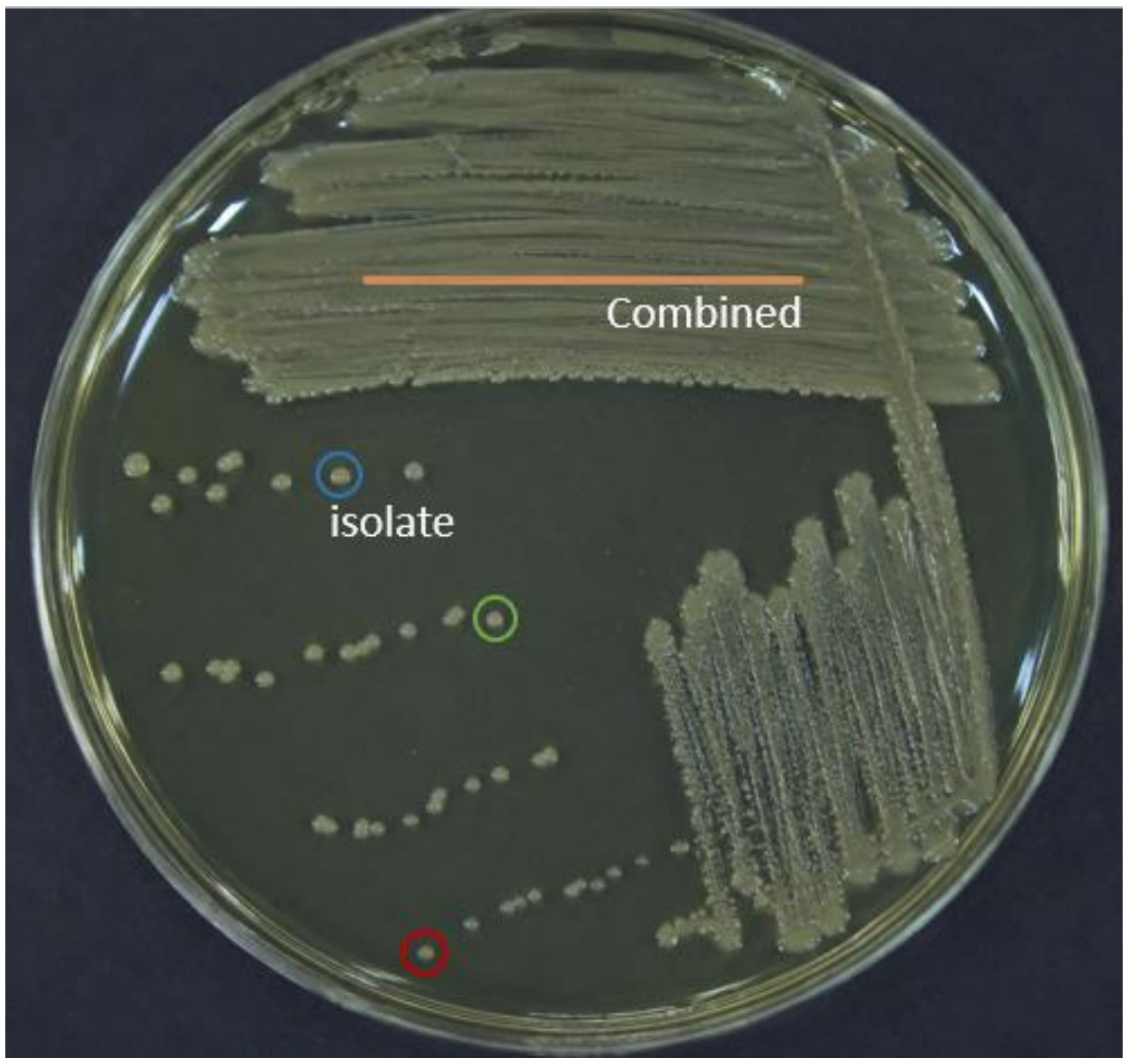

Figure S7. Schematic representation of isolated colonies and combined *E. coli* populations. Isolate populations 1-3 each represent cells collected from a separate, well-isolated colony. The combined population represents cells collected from the confluent growth region. For each experiment, one isolate or combined population was picked from a freshly prepared plate on the same day the experiment was performed.

Table S1. Summary of single-cell kinetic parameters extracted from PI fluorescence trajectories during aPDT.

bacteria	isolates[a]	t_i range (min)	t_f range (min)	Δt range (min)[b]	t_f vs t_i slope[c]	t_f vs Δt slope[c]	t_f vs Δt intercept (min)[c]
E. coli	1	169.1	180.8	82.3	1.66 ± 0.04	1.69 ± 0.11	21.17 ± 5.00
	2	164.6	218.5	95.0	1.55 ± 0.03	1.39 ± 0.16	68.79 ± 10.19
	3	139.1	211.35	94.5	1.40 ± 0.02	1.75 ± 0.10	35.66 ± 3.10
	combined	188.7	268.0	119.3	1.58 ± 0.02	2.13 ± 0.09	23.26 ± 6.28
S. aureus	1	6.8	24.5	26.1	6.35 ± 0.47	0.92 ± 0.02	1.95 ± 0.18
	2	10.5	46.0	43.6	2.87 ± 0.77	0.98 ± 0.02	0.90 ± 0.45
	3	12.1	38.1	36.5	6.45 ± 0.41	0.93 ± 0.02	4.50 ± 0.47
	combined	7.1	55.2	51.3	8.44 ± 0.51	1.01 ± 0.02	1.77 ± 0.51

**bacteria**	**isolates**[a]	***ti* range (min)**	**t_f range (min)**	**Δt range (min)**[b]	**t_f vs t_i slope**[c]	**t_f vs Δt slope**[c]	**t_f vs Δt intercept (min)**[c]
E. coli	1	104.0	223.2	214.62	2.12 ± 0.07	1.13 ± 0.04	40.70 ± 2.84
	2	141.7	261.6	276.93	2.46 ± 0.10	1.17 ± 0.04	25.46 ± 3.99
	3	185.1	259.4	180.67	1.79 ± 0.04	1.36 ± 0.08	55.27 ± 6.42
	combined	322.5	425.5	267.58	1.77 ± 0.04	1.00 ± 0.08	141.40 ± 12.19
S. aureus	1	5.1	40.5	30.51	12.08 ± 0.52	1.01 ± 0.02	1.61 ± 0.55
	2	14.9	66.8	63.24	8.05 ± 0.40	1.00 ± 0.02	3.88 ± 0.90
	3	12.1	73.7	76.52	10.43 ± 0.47	1.03 ± 0.02	2.95 ± 1.29
	combined	15.7	60.5	58.61	4.34 ± 0.11	1.05 ± 0.02	7.95 ± 0.66

[a]The isolate numbers are condition-specific labels and do not denote the same single-colony-derived population across different media or species.
[b]Δt range = longest and shortest PI-transition durations measured among the individual cells in that population.
[c]Regression values are reported as fit parameter ± standard error. Each row summarizes kinetic parameters extracted from 150 individual cells for the corresponding combination of species, medium, and population condition.

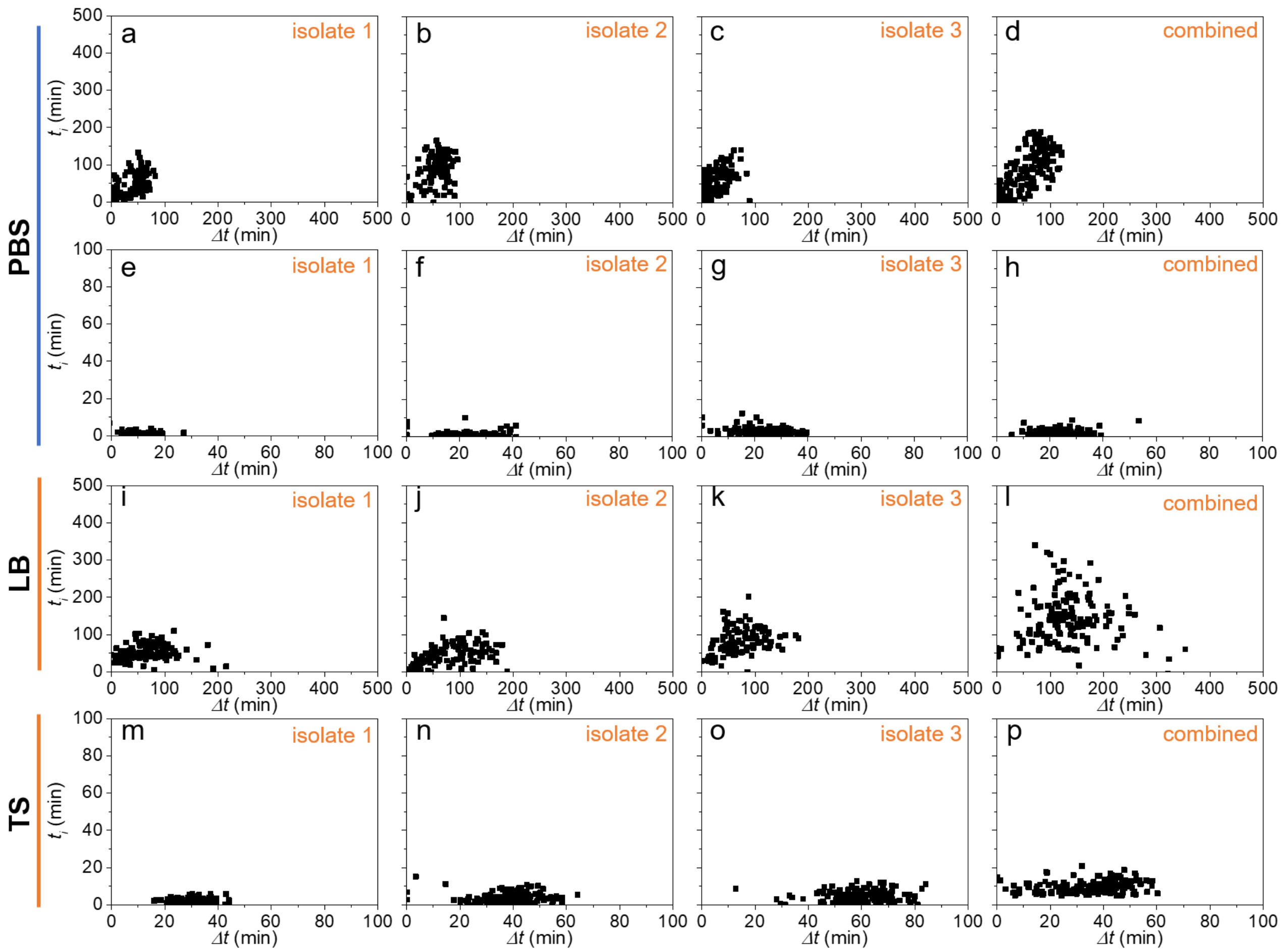

Figure S8. Relationship between t_i and Δt across isolate-derived and combined populations. Scatter plots show the relationship between *ti* and Δt for individual bacterial cells during aPDT. Panels (a-d) correspond to *E. coli* in PBS, panels (e-h) to *S. aureus* in PBS, panels (i-l) to *E. coli* in LB, and panels (m-p) to *S. aureus* in TS. The power of excitation light was 0.66 ± 0.02 mW. Light dose varied between 4.75 ± 0.14 and 14.3 ± 0.4 J/cm^2.

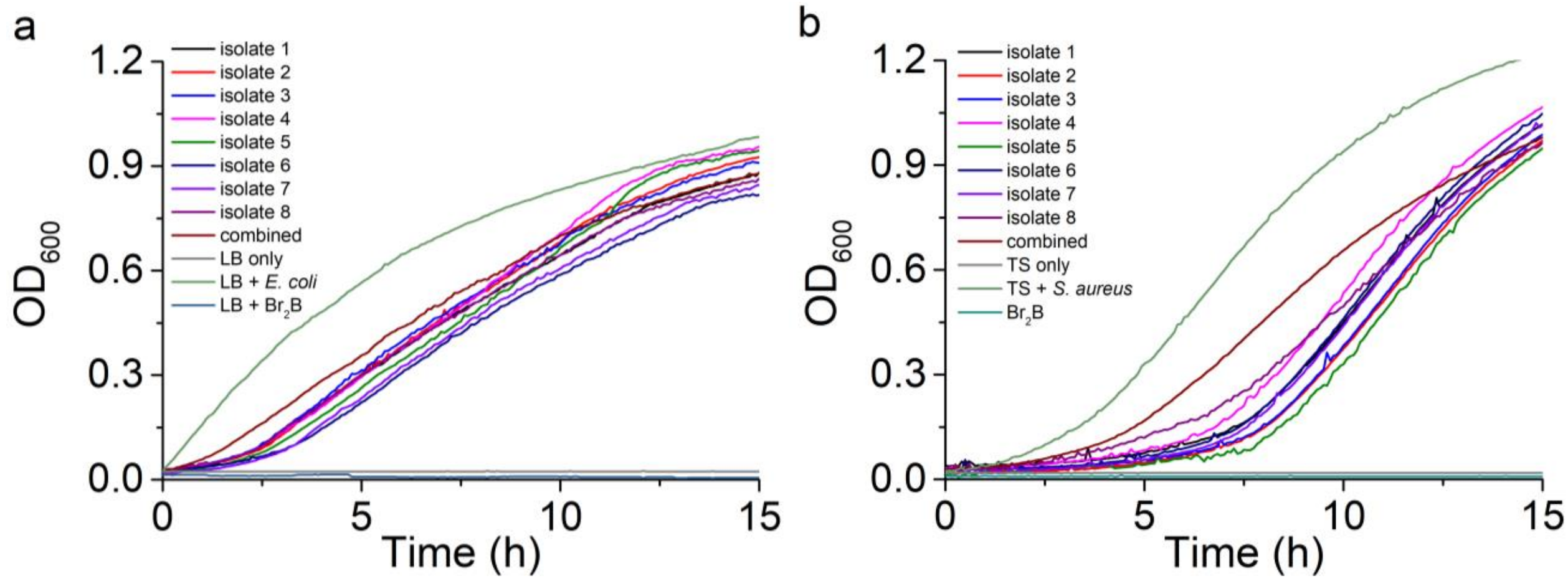

Figure S9. Post-aPDT regrowth of isolate-derived and mixed-founder bacterial populations. OD_{600} growth curves were recorded for 15 h after Br_2B-mediated photodynamic treatment. (a) *E. coli* populations in LB, including eight single-colony-derived isolates (isolate 1-8), the combined population, and LB-only, LB + *E. coli*, and LB + Br_2B controls. (b) *S. aureus* populations in TS, including eight single-colony-derived isolates (isolate 1-8), the combined mixed-founder population, and TS-only, TS + *S. aureus*, and TS + Br_2B controls. Light dose: 7.74 ± 0.4 J/cm^2 and 3.87 ± 0.4 J/cm^2 for *E. coli* and *S. aureus*, respectively.

Analysis of bacterial Growth Curves using the modified Gompertz equation

Equation. The modified Gompertz model is widely used to analyze bacterial growth curves because it provides biologically interpretable parameters for the lag phase, maximum growth rate, and asymptotic growth yield.[4]

For baseline-corrected growth curves, the three-parameter modified Gompertz model is:

$$y(t) = A \cdot \exp\left\{-\exp\left[\frac{\mu_{max} \cdot e}{A}(\lambda - t) + 1\right]\right\}$$

where:

y(t): baseline-corrected OD_{600} at time *t*.

A: asymptotic growth yield, expressed as the net OD600 increase above baseline.

μ_{max}: maximum growth rate, expressed in OD_{600}/h.

λ: lag phase duration, expressed in hours.

e: Euler's number.

Fittings for *E. coli*.

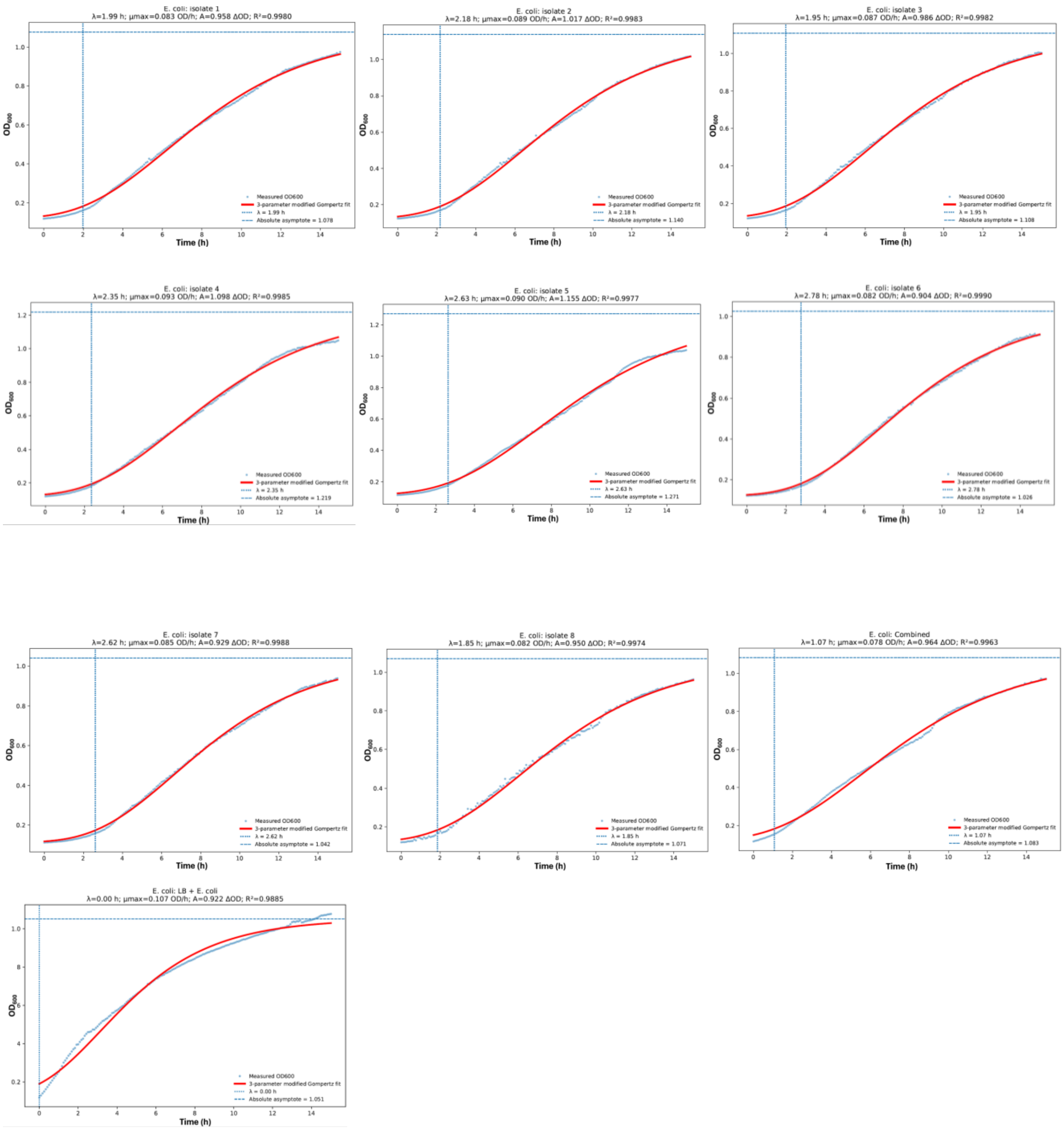

Figure S10. Modified Gompertz fits for post-aPDT regrowth of *E. coli* populations. Representative OD_{600} growth curves for *E. coli* isolate-derived populations and the combined mixed-founder population were fitted using the three-parameter modified Gompertz model. Black points represent the experimental data, and red lines represent the fitted Gompertz curves. The vertical dashed blue line indicates the fitted λ, defined as the time required before detectable population regrowth. The fits were also used to extract μ_{max} and A.

Fitting for *S. aureus*.

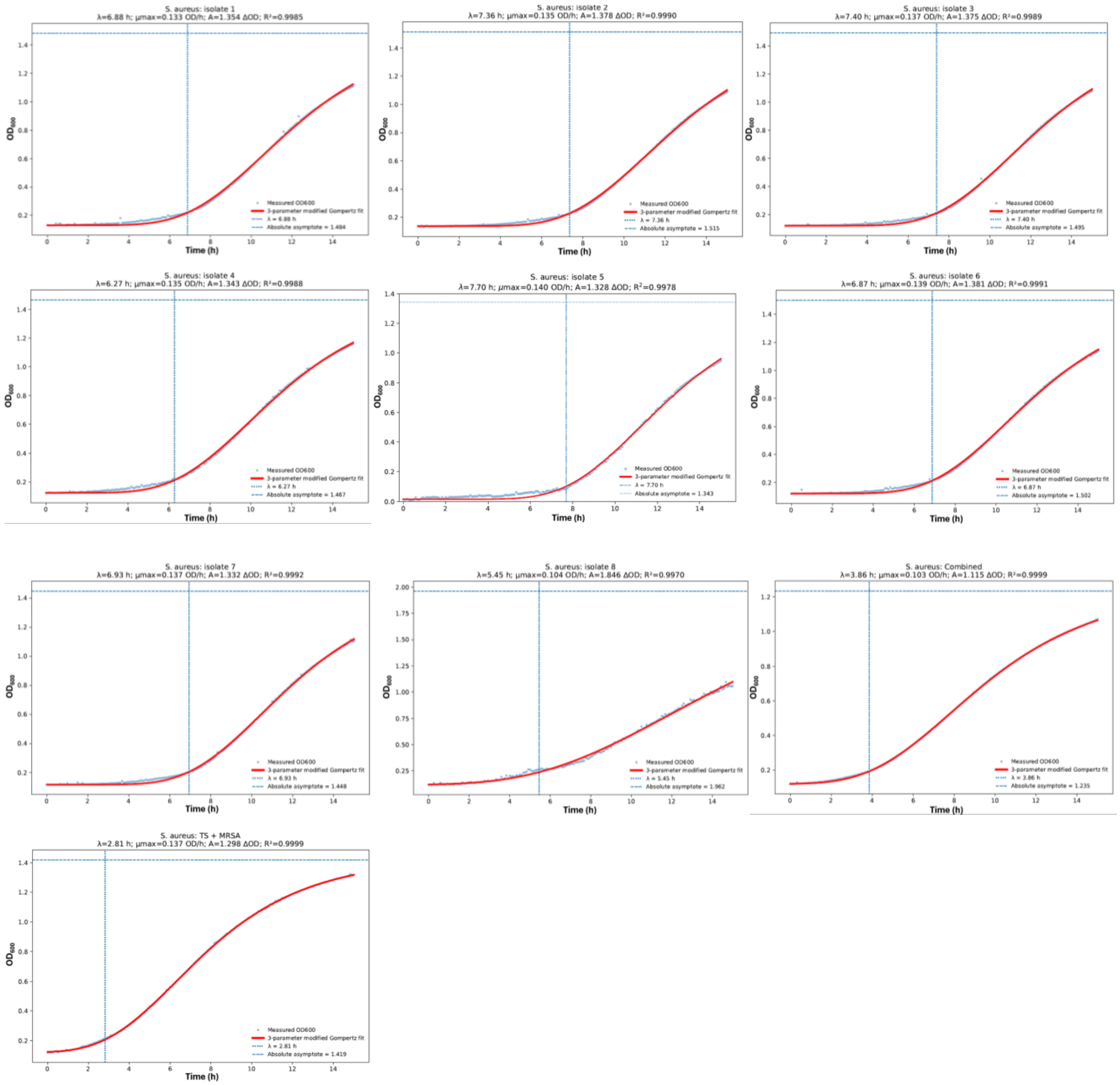

Figure S11. Modified Gompertz fits for post-aPDT regrowth of *S. aureus* populations. Representative OD_{600} growth curves for *S. aureus* isolate-derived populations and the combined mixed-founder population were fitted using the three-parameter modified Gompertz model. Black points represent the experimental data, and red lines represent the fitted Gompertz curves. The vertical dashed blue line indicates the fitted λ, defined as the time required before detectable population regrowth. The fits were also used to extract μ_{max} and A.

Table S2. Modified Gompertz parameters extracted from post-aPDT bulk regrowth curves after baseline correction.

species	population	*λ (h)*	μ_{max} *(OD₆₀₀/h)*	*A*
E. coli	isolate 1	1.992	0.083	0.958
	isolate 2	2.180	0.089	1.017
	isolate 3	1.950	0.087	0.986
	isolate 4	2.348	0.093	1.098
	isolate 5	2.632	0.090	1.155
	isolate 6	2.779	0.082	0.904
	isolate 7	2.616	0.085	0.929
	isolate 8	1.853	0.082	0.950
	combined	1.072	0.078	0.964
	untreated	0.000	0.107	0.922
S. aureus	isolate 1	6.878	0.133	1.354
	isolate 2	7.355	0.135	1.378
	isolate 3	7.402	0.137	1.375
	isolate 4	6.274	0.135	1.343
	isolate 5	7.700	0.140	1.328
	isolate 6	6.867	0.139	1.381
	isolate 7	6.934	0.137	1.332
	isolate 8	5.453	0.104	1.846
	combined	3.862	0.103	1.115
	untreated	2.808	0.137	1.298

Calculation to estimate mutational heterogeneity in single-colony and mixed-colonies

Assumptions. Genome sizes were treated as the number of mutational sites for final fixed-mutation calculations. *E. coli* UMN026 was modeled as 5,358,200 bp total DNA, based on a 5,202,090 bp chromosome plus plasmids of 122,301 bp and 33,809 bp. Plasmids were treated as single-copy targets for this simplified estimate.[5] *S. aureus* SA113 was modeled as 2,829,793 bp/sites. Final fixed mutation rates were expressed as mutations per nucleotide per generation.[6,7]

Each colony was assumed to arise from one founder cell with no selection among mutant lineages. The estimates refer to newly acquired mutations arising during colony expansion relative to the colony founder and do not include mutations already present in the freezer stock, inoculum, or pre-

culture. The calculations are order-of-magnitude estimates and not direct mutation-accumulation measurements for the specific laboratory stocks used in the experiments.

Equations.

Final fixed mutation-rate (μ_{genome}) equations:

$$\mu_{\text{genome}} = \mu_{\text{nt}} \times L_{\text{genome}}$$

$$L_{E.\,coli} = 5.36 \times 10^{6}\ \text{bp}$$

$$\mu_{\text{nt},E.\,coli} = 2.20 \times 10^{-10}\ \text{mutations per nucleotide per generation}$$

$$\mu_{\text{genome},E.\,coli} = (5.36 \times 10^{6}\ \text{bp})(2.20 \times 10^{-10}\ \text{mutations per nucleotide per generation})$$

$$\mu_{\text{genome},E.\,coli} = 1.18 \times 10^{-3}\ \text{mutations per nucleotide per generation}$$

$$\frac{1}{1.18 \times 10^{-3}} \approx 8.5 \times 10^{2}\ \text{generations per mutation}$$

$$L_{S.\,aureus} = 2.83 \times 10^{6}\ \text{bp}$$

$$\mu_{\text{genome},S.\,aureus} = (2.83 \times 10^{6}\ \text{bp})(2.70 \times 10^{-10}\ \text{mutations per nucleotide per generation})$$

$$\mu_{\text{genome},S.\,aureus} = 7.64 \times 10^{-4}\ \text{mutations per nucleotide per generation}$$

$$\frac{1}{7.64 \times 10^{-4}} \approx 1.3 \times 10^{3}\ \text{generations per mutation}$$

Colony expansion equations (estimate of 3.3×10^{9} cells per colony):

$$N = 2^{g}$$

$$g = \log_{2}(N) = \frac{\ln(N)}{\ln(2)}$$

$$g = \log_{2}(3.3 \times\ 10^{9}) = 31.6$$

Probability (P) that a terminal cell lineage acquired at least one mutation:

$$P = 1 - e^{-\mu_{\text{genome}} g}$$

$$P_{E.\,coli} = 1 - e^{-(1.2 \times 10^{-3})(31.6)}$$

$$P_{E.\,coli} = 1 - e^{-0.038} \approx 0.037 \approx 3.7\%$$

$$P_{S.\,aureus} = 1 - e^{-(7.6 \times 10^{-4})(31.6)}$$

$$P_{S.\,aureus} = 1 - e^{-0.024} \approx 0.024 \approx 2.4\%$$

Expected number of terminal cells carrying at least one mutation (N_{mutated}):

$$N_{\text{mutated}} = N_{\text{total}} \times P(\geq 1 \text{ mutation})$$

$$N_{\text{mutated},E.\,coli} = (3.3\ \times 10^{9})(0.037) = 1.2 \times 10^{8} \text{ cells}$$

$$N_{\text{mutated},S.\,aureus} = (3.3\ \times 10^{9})(0.024) = 7.8 \times 10^{7} \text{ cells}$$

Expected total number of mutation events (M_{events}) during colony expansion:

$$M_{\text{events}} = \mu_{\text{genome}} \times N_{\text{total divisions}}$$

$$N_{\text{total divisions}} \approx N_{\text{final}} - 1$$

$$M_{\text{events}} \approx \mu_{\text{genome}} \times N$$

$$M_{\text{events},E.\,coli} \approx (1.18 \times 10^{-3})(3.3 \times 10^{9}) = 3.90 \times 10^{6} \text{ events}$$

$$M_{\text{events},S.\,aureus} \approx (7.64 \times 10^{-4})(3.3 \times 10^{9}) = 2.52 \times 10^{6} \text{ events}$$

Envelope/cell-wall mutation equations:

$$P_{env} = 1 - e^{-\mu_{genome}\, g\, f_{env}}$$

$$f_{env} = 0.01 - 0.05$$

Where f_{env} denotes the fraction of genome-wide mutational target space represented by envelope or cell wall associated genes and loci.

$$P_{env,E.coli} = 1 - e^{-(1.18 \times 10^{-3})(31.6)(0.01-0.05)}$$

$$P_{env,E.coli} = 3.79 \times 10^{-4} - 1.90 \times 10^{-3}$$

$$P_{env,E.coli} = 0.038\% - 0.190\%$$

$$P_{env,S.aureus} = 1 - e^{-(7.64 \times 10^{-4})(31.6)(0.01-0.05)}$$

$$P_{env,S.aureus} = 2.40 \times 10^{-4} - 1.20 \times 10^{-3}$$

$$P_{env,S.aureus} = 0.024\% - 0.120\%$$

References

(1) Schindelin, J.; Arganda-Carreras, I.; Frise, E.; Kaynig, V.; Longair, M.; Pietzsch, T.; Preibisch, S.; Rueden, C.; Saalfeld, S.; Schmid, B.; et al. Fiji: an open-source platform for biological-image analysis. *Nat. Methods* **2012**, *9*, 676-682.
(2) Etheridge, T. J.; Carr, A. M.; Herbert, A. D. GDSC SMLM: Single-molecule localisation microscopy software for ImageJ. Wellcome Open Research **2022**, *7*, 241.
(3) Martínez, S. R.; Caverzan, M.; Ibarra, L. E.; Aiassa, V.; Bohl, L.; Porporatto, C.; Gómez, M. L.; Chesta, C. A.; Palacios, R. E. Light-activated conjugated polymer nanoparticles to defeat pathogens associated with bovine mastitis. *J. Photochem. Photobiol. B, Biol.* **2024**, *257*, 112971.
(4) Tjørve, K. M. C.; Tjørve, E. The use of Gompertz models in growth analyses, and new Gompertz-model approach: An addition to the Unified-Richards family. *PLOS ONE* **2017**, *12*, e0178691.
(5) Lescat, M.; Calteau, A.; Hoede, C.; Barbe, V.; Touchon, M.; Rocha, E.; Tenaillon, O.; Médigue, C.; Johnson, J. R.; Denamur, E. A module located at a chromosomal integration hot spot is responsible for the multidrug resistance of a reference strain from *Escherichia coli* clonal group A. *Antimicrob. Agents Chemother.* **2009**, *53*, 2283-2288.
(6) Lee, H.; Popodi, E.; Tang, H.; Foster, P. L. Rate and molecular spectrum of spontaneous mutations in the bacterium *Escherichia coli* as determined by whole-genome sequencing. *Proc. Natl. Acad. Sci.* **2012**, *109*, E2774-E2783.
(7) American Type Culture Collection (ATCC). Staphylococcus aureus subsp. aureus (ATCC® 35556™): Genome Assembly Summary. *ATCC Genome Portal.* Published March 27, 2020. Accessed June 22, 2026.
